# Reinfection in patients with COVID-19: a systematic review

**DOI:** 10.1186/s41256-022-00245-3

**Published:** 2022-04-29

**Authors:** Xiangying Ren, Jie Zhou, Jing Guo, Chunmei Hao, Mengxue Zheng, Rong Zhang, Qiao Huang, Xiaomei Yao, Ruiling Li, Yinghui Jin

**Affiliations:** 1 Center for Evidence-Based and Translational Medicine, Zhongnan Hospital of Wuhan University, Wuhan, China; 2 College of Nursing and Health, Henan University, Kaifeng, Henan China; 3 School of Nursing, Wuhan University, Wuhan, China; 4 Department of Acupuncture Rehabilitation, The Affiliated Hospital of Nanjing University of Chinese Medicine, Nanjing, China; 5 The First Clinical College of Wuhan University, Wuhan, Hubei China; 6 Department of Neurotumor Disease Diagnosis and Treatment Center, Taihe Hospital, Hubei University of Medicine, Shiyan, China; 7 Department of Health Research Methods, Evidence, and Impact, McMaster University, Hamilton, ON Canada; 8 Center for Clinical Practice Guideline Conduction and Evaluation, Children’s Hospital of Fudan University, Shanghai, China

**Keywords:** COVID-19, Reinfection, Systematic review

## Abstract

**Background:**

With the continuation of the COVID-19 pandemic, some COVID-19 patients have become reinfected with the virus. Viral gene sequencing has found that some of these patients were reinfected by the different and others by same strains. This has raised concerns about the effectiveness of immunity after infection and the reliability of vaccines. To this end, we conducted a systematic review to assess the characteristics of patients with reinfection and possible causes.

**Methods:**

A systematic search was conducted across eight databases: PubMed, Embase, Web of Science, The Cochrane Library, CNKI, WanFang, VIP and SinoMed from December 1, 2019 to September 1, 2021**.** The quality of included studies were assessed using JBI critical appraisal tools and Newcastle–Ottawa Scale.

**Results:**

This study included 50 studies from 20 countries. There were 118 cases of reinfection. Twenty-five patients were reported to have at least one complication. The shortest duration between the first infection and reinfection was 19 days and the longest was 293 days. During the first infection and reinfection, cough (51.6% and 43.9%) and fever (50% and 30.3%) were the most common symptoms respectively. Nine patients recovered, seven patients died, and five patients were hospitalized, but 97 patients’ prognosis were unknown. B.1 is the most common variant strain at the first infection. B.1.1.7, B.1.128 and B.1.351 were the most common variant strains at reinfection. Thirty-three patients were infected by different strains and 9 patients were reported as being infected with the same strain.

**Conclusions:**

Our research shows that it is possible for rehabilitated patients to be reinfected by SARS-COV-2. To date, the causes and risk factors of COVID-19 reinfection are not fully understood. For patients with reinfection, the diagnosis and management should be consistent with the treatment of the first infection. The public, including rehabilitated patients, should be fully vaccinated, wear masks in public places, and pay attention to maintaining social distance to avoid reinfection with the virus.

**Supplementary Information:**

The online version contains supplementary material available at 10.1186/s41256-022-00245-3.

## Introduction

As COVID-19 epidemic continues to spread worldwide, it has caused 263,563,622 confirmed cases of COVID-19, including 5,232,562 deaths as of 3 December 2021 [1]. Severe acute respiratory syndrome coronavirus 2 (SARS-CoV-2) is a single-stranded positive-strand RNA virus that belongs to the Coronaviridae family [2, 3]. Coronaviruses (CoVs) were previously known to be present in the environment and to infect humans, for example SARS-CoV and Middle East respiratory syndrome coronavirus (MERS-CoV) have appeared in the past two decades. SARS-CoV-2 is characterized by efficient transmission despite having a lower mortality rate compared with the other two CoVs [4]. A number of animal experiments have shown reinfection with the same or a different strain after initial infection with SARS-CoV-2 for more than or equal to 21 [5, 6] and 28 days [7]. This suggests that humans can also be at risk of being reinfected.

In fact, reinfected people have been reported during the present outbreak. The first case of COVID-19 reinfection was described in Hong Kong in August 2020, a thirty-three years old male was asymptomatic during the second infection and different strains of SARS-CoV-2 were identified in the two infections [8]. Subsequently, many countries, such as the United States [9] and Italy [10], have also reported the emergence of reinfected patients.

The SARS-CoV-2 continues to mutate, and new mutations have appeared in the Netherlands [11], the United States [12], India [13] and elsewhere. World Health Organization (WHO) has announced new easy-to-remember labels for Variants of Interest (VOIs) and Variants of Concern (VOC) to facilitate public communication about SARS-CoV-2 variants, these currently include Alpha (B.1.1.7), Beta (B.1.351), Gamma (P.1), Delta (B.1.617.2) and Omicron (B.1.1.529) [14]. The emergence of a variant may affect the retransmission of the disease, its severity and doctors’ ability to diagnose, treat, prevent, and control the infection [15, 16]. However, studies have shown that compared to other variants, the Omicron variants pose an increased risk of reinfection [17]. It has also caused public concern and controversy, which includes questions about the contagious nature of reinfected patients, the effectiveness of vaccines and their usefulness against virus variants. Knowing the frequency and natural course of reinfections is important for developing strategies to control SARS-CoV-2.

Many studies have defined re-positive RT-PCR as reinfection which may not always be the case, or have not reported viral gene sequencing results or have omitted clear epidemiological data of patients with reinfections, which will greatly distort the description of the number and characteristics of reinfected patients. Knowledge about reinfected patients is still inadequate and limited. Therefore, because of the need to target confirmed reinfections in patients we have done this review in order to provide clear information for this paper. The present study provides an independent definition of reinfected persons: laboratory confirmation of two infections with the same or different virus strains by lineage, clades, phylogenetic analysis (proof of two distinct virus variants with any sequence variation between the two episodes) for the first and second infections. If there are no laboratory data on the first infection, clear epidemiological data are needed (eg. there are clear epidemiological data to indicate that the virus reinfecting the patient was not spreading locally at the time of the patient's initial infection, so as to prove that the virus strains of the two infections are unrelated).

The purpose of this systematic review is to summarize the characteristics of patients with proven reinfection, including details of clinical symptoms, viral load, and viral gene sequencing of primary infections and subsequent reinfections, and whether or not these patients are contagious. In addition, we will discuss the potential reasons for reinfection to provide advice on management of reinfected patients.

## Methods

The study protocol was registered at PROSPERO, which is an ongoing systematic review registry (ID: CRD42021265333) [18]. This review was performed and reported in accordance with Preferred Reporting Items for Systematic Reviews and Meta-analyses 2020 (PRISMA 2020) [19].

### Data sources and search strategy

We searched the following eight databases: PubMed, Embase, Web of Science, The Cochrane Library, CNKI, WanFang, VIP and SinoMed from December 1, 2019 to September 1, 2021. At the same time, we checked the previous relevant systematic reviews on the topic to ensure that no eligible articles were missed [20–27]. We constructed a detailed search strategy to fully capture the reinfected patients, and Additional file 1: Table S1 provides the search strategy for databases. We applied no restrictions for language of publications. Studies were selected for further consideration through screening of titles, abstracts, and methods for relevance based on the eligibility criteria after excluding duplications. Two independent researchers (XY Ren and J Zhou) screened retrieved articles and both of them reviewed each article. These investigators then independently assessed full texts of records deemed eligible for inclusion. Any discrepancies were resolved by discussion with other co-authors.

### Eligibility criteria

Studies were selected based on the following inclusion criteria: (1) papers recruited patients that met our definition of reinfection; (2) reported outcomes of interest included description of clinical symptoms of both infections, viral gene sequencing, virus load, or infectivity; (3) original research with any type of observational study (cohort study, cross-sectional study, case–control study, case report and case series).

Exclusion criteria are: (1) articles focusing on animal experiments; (2) Full texts of studies were not available.

### Data extraction

Two independent reviewers (XY Ren and J Zhou) extracted data from each eligible study and then cross-checked the results. Disagreements between reviewers regarding extracted data were resolved through discussion and consensus with the third reviewer (J Guo). We extracted data about the constructed indices from all papers that met the inclusion criteria, which included first author name, date of publication, country, type of study, age, sex and co-morbidities of the reinfected patients, the proportion of reinfected patients among discharged patients, the time interval between the first and second clinical symptoms, results of virus gene sequencing and the cycle threshold (Ct) value of both infections, vaccination status, and the patient outcomes.

### Quality assessment

Included articles were independently assessed for quality by two reviewers (CM Hao and MX Zheng) using criteria based on the standard principles of quality assessment. The methodological quality of the included case reports, case series, cross-sectional and case–control studies were assessed based on JBI critical appraisal tools [28]. The quality of each checklist item was graded as Yes, No, Unclear or Not applicable. The methodological quality for the cohort studies was assessed based on Newcastle–Ottawa Scale [29]. The quality was ranked as: unsatisfactory (0–4 points), satisfactory (5–6 points), and good (7–8 points), or very good (9–10 points) [30]. The three reviewers then shared the quality assessment checklist results and reached consensus through discussion.

## Results

### Search results

**Fig. 1:**
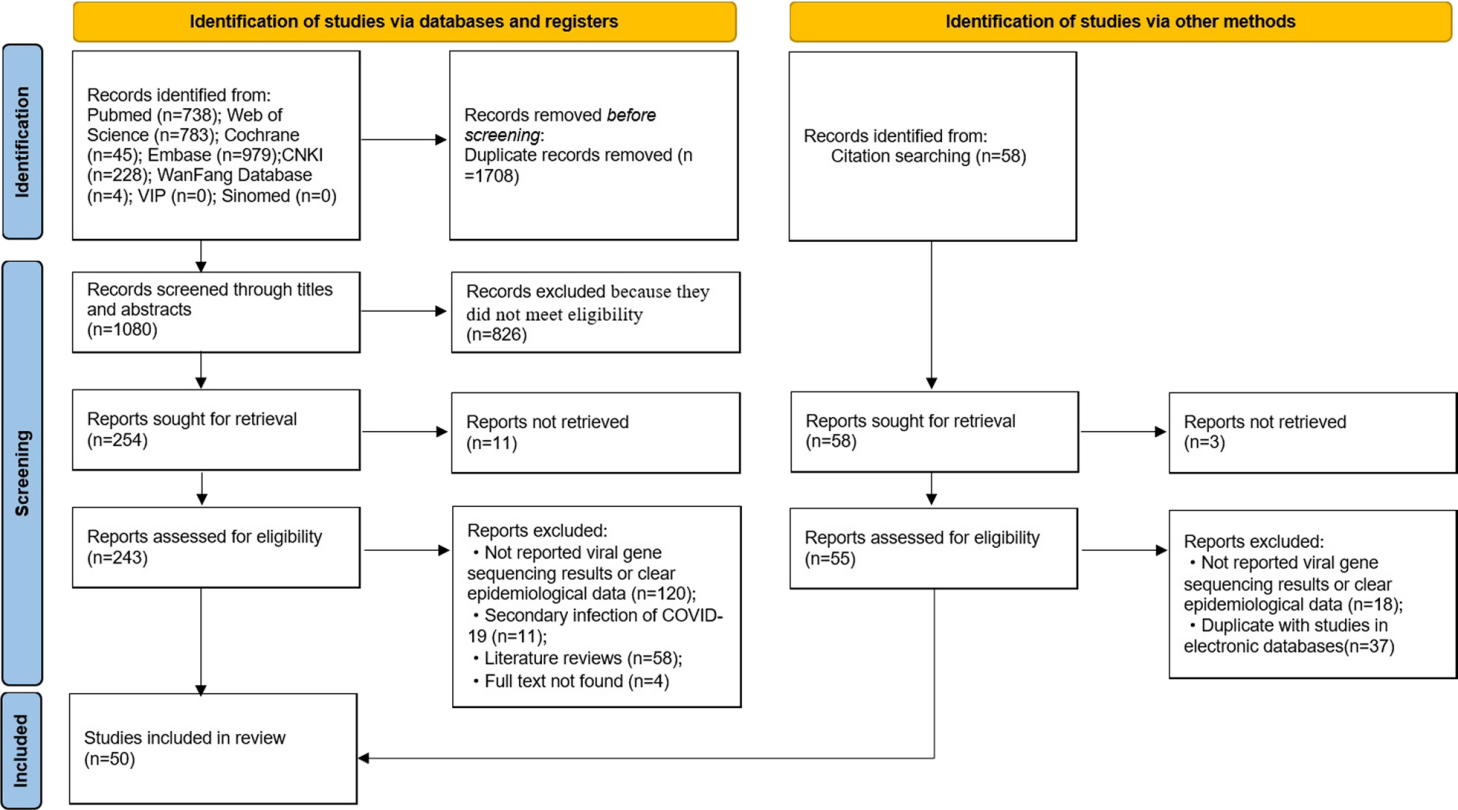
PRISMA flow chart to show the study selection process

A total of 2788 records were identified in the initial literature search. After removing 1708 duplicates, 1080 articles were screened by titles and abstracts, and 837 articles were excluded. 243 studies were reviewed using the full texts and finally 50 articles met the inclusion criteria and were analyzed in the systematic review (Fig. 1). In these studies, there were 46 case reports [8–10, 31–73], 2 cross-sectional studies [74, 75], 1 cohort study [76] and 1 case–control study [77]. Ten papers were from Brazil, 7 from the United States, 5 from India, 4 from Italy, 3 from the United Kingdom, 12 studies, 2 each from Spain, Belgium, Ecuador, Netherlands, Iran and France. The remaining 9 studies came from Panama, Qatar, Luxembourg, South Korea, Saudi Arabia, Switzerland, Colombia, Germany and China.

### Study quality assessment

Overall, the methodological quality of 46 case reports (Additional file 1: Table S2) and 1 cohort study (Additional file 1: Table S5) were moderate to high, 1 case–control study (Additional file 1: Table S4) was moderate because it did not identify and deal with the confounding factors. The methodological quality of 2 cross-sectional studies were moderate (Additional file 1: Table S3) because neither of them had clear exposure factors.

### Characteristics of reinfected patients

**Fig. 2:**
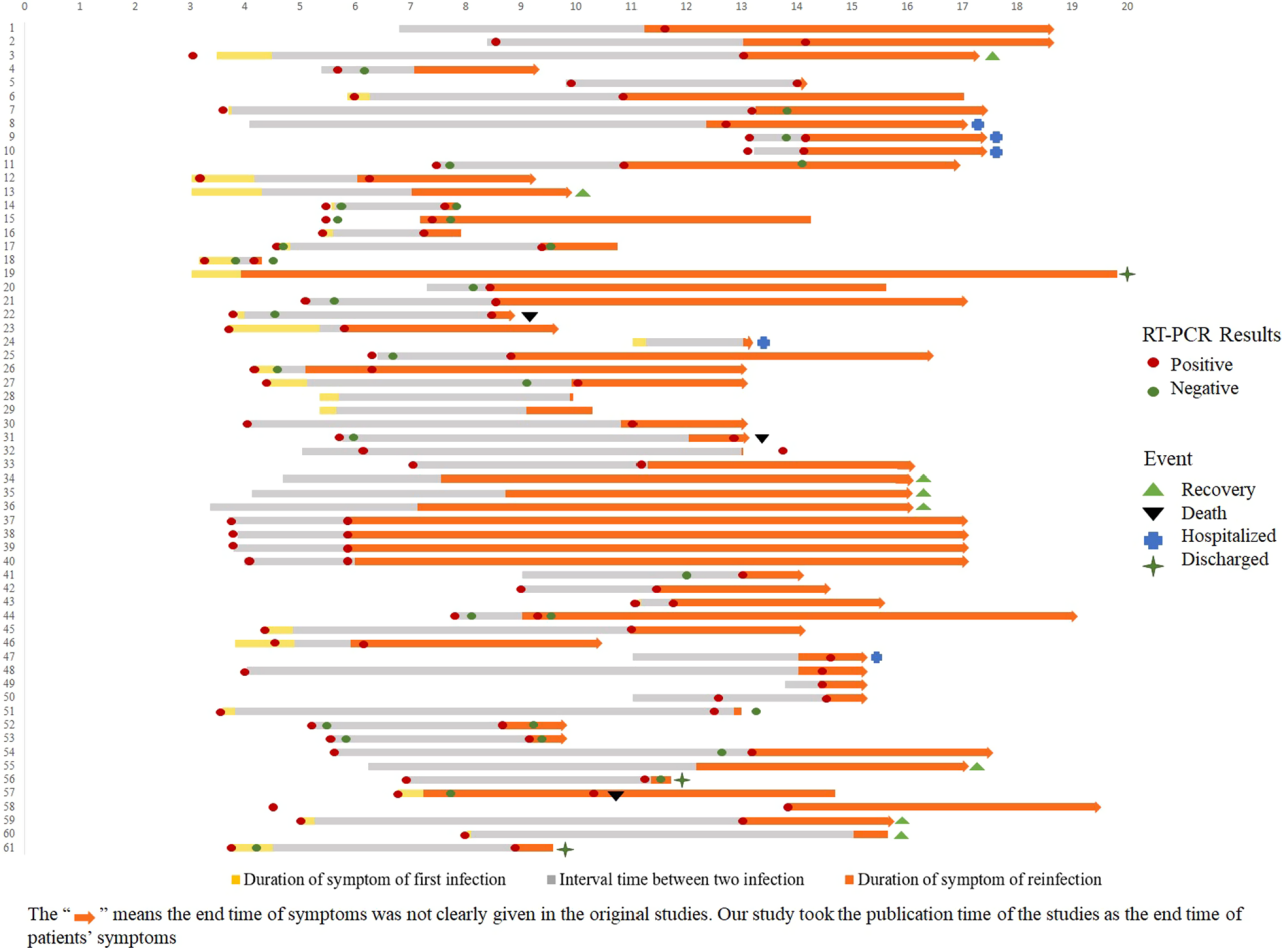
Duration of symptom of two infection

A total of 118 reinfected patients were included in 50 studies. These reinfected patients have a wide age distribution (a range of 16–92 years), with a gender distribution of 62 (52.5%) male and 54 (45.8%) female (two case reports did not report gender), including 24 healthcare staff (9 male and 15 female). 25 patients were reported as having at least one comorbidity (such as hypertension, end-stage renal disease, asthma.). Patients often presented with overt symptoms upon reinfection. Characteristics of reinfected patients are presented in Table 1. Figure 2 shows the duration of symptoms between the two infections and outcomes in reinfected patients. The corresponding patient information in Fig. 2 is shown in the Additional file 1: Table S6.

**Table 1:** Characteristics of the included studies (a) Part 1 and (b) Part 2

Study	Country	Study type	Reinfected patients (gender, age)	Reinfected patients/re-positive patients	Time between episodes (days, month)	Clinical symptoms^#^	
						1st episode	2nd episode
**(a) Part 1**							
To et al. [8]	China	Case report	M, 33	–	142d	Cough and sputum, sore throat, fever and headache	Asymptomatic
Tillett et al. [9]	United States	Case report	M, 25	–	65d	Sore throat, cough, headache, nausea, and diarrhoea	Myalgia, cough, and shortness of breath
Borgogna et al. [10]	Italy	Case report	M, 52	–	–	Cough and fever	–
Gupta et al. [31]	India	Case report	M, 25 (HCW)	–	108d	Asymptomatic	Asymptomatic
			F,28 (HCW)	–	111d	Asymptomatic	Asymptomatic
Larson et al. [32]	United States	Case report	M, 42	–	51d	Cough, subjective fever, and myalgia	Fevers, cough, shortness of breath and gastrointestinal symptoms
Staub et al. [33]	Luxembourg	Case report	M, 20s (HCW)	–	4m	Loss of smell and taste	Cough
			F, 20s (HCW)	–	11m	Fever, headache, chills, diarrhoea, loss of taste and smell	Fever, chills, and headache
			M, 30s (HCW)	–	20d	Asymptomatic	Chills, myalgia, and headache
			F, 20s (HCW)	–	4m	Fever, muscle pain, headache, loss of taste and smell	Muscle pain and cough
Salehi-Vaziri et al. [34]	Iran	Case report	F, 32	–	63d	Headache, sore throat, cough, fever	Severe cough, fever, fatigue (severe)
			M, 54		156d	Fatigue, anxiety, chest pain, cough, fever	Milder fatigue, chest pain, dizziness, diarrhea(less)
			M, 42		111d	Shortness of breath, sore throat, shaking chills, pain, diarrhea	Similar to the first infection with severe diarrhea (similar)
Klein et al. [35]	United States	Case report	M, 60–70 (specific age unknown	–	232d	Fevers, fatigue, and dry cough	Fatigue and nonproductive cough
Shastri et al. [36]	India	Case report	M, 27 (HCW)	–	64d	Sore throat, nasal congestion and rhinitis	Myalgia, fever, non-productive cough, fatigue
			F, 24 (HCW)		52d	Sore throat, rhinitis and myalgia	Fever, myalgia, rhinitis, sore throat, non-productive cough and fatigue
			F, 25 (HCW)		136d	Fever, myalgia, dry cough.	Fever, myalgia, dry cough, nausea, abdominal pain, breathlessness on exertion.
Vetter et al. [37]	Switzerland	Case report	F, 36	–	200d	Asthenia and headache	Asthenia, shivering, rhinorrhoea, anosmia, arthralgia, headache and exertional dyspnoea
Harrington et al. [38]	United Kingdom	Case report	M, 78	–	253d	Mild illness	Shortness of breath, severe hypoxia (severe)
Prado-Vivar et al. [39]	Ecuador	Case report	M, 46	–	72d	Intense headache and drowsiness	Odynophagia, nasal congestion, fever of 38.5°C, strong back pain, productive cough and dyspnea (severe)
Moschetta et al. [40]	Brazil	Case report	F.39	–	8m	fever and cough	headache, myalgia, fever, and cough
			M, 49	–	7m	cough with sputum	fever and cough
Scarpati et al. [41]	Italy	Case report	M, 63(HCW)	–	–	Asymptomatic	Respiratory failure. fever
Massanella et al. [42]	Spain	Case report	M, 62 (HCW)	–	–	fever, diarrhea, anosmia, dysgeusia, cough, intense asthenia, and arthromyalgias	intense arthromyalgias, headache, fever, cough, and dyspnea
Garvey et al. [43]	United Kingdom	Case report	M, 92	–	206d	Pyrexia, a dry cough and shortness of breath	Lethargy, persistent cough and pyrexia
			M, 84		224d	Lethargy and confusion	Asymptomatic
			M, 59		236d	Cough and fluctuating temperature	Asymptomatic
Siqueira et al. [44]	Brazil	Case report	F, 76	–	105d	Cough and fever	–
Sevillano et al. [45]	Ecuador	Case report	M, 28	–	104d	Sore throat, cough, headache, nausea, and diarrhea, anxiety and panic attacks	Anosmia, ageusia, fever, headache
Kulkarni et al. [46]	India	Case report	M, 61	–	103d	Asymptomatic	Weakness, cough
Lee et al. [47]	South Korea	Case report	F, 21	1/4	32d	Sore throat and cough (mild)	Sore throat and productive cough
Fintelman-Rodrigues et al. [48]	Brazil	Case report	M, 54	–	65d	Headache	Fever, dry cough, tiredness, body ache, anosmia, ageusia
			M, 34		63d	Asymptomatic	Fever, nausea, tiredness, headache, body ache
			F, 57		61d	Mild diarrhea	Fever, diarrhea, headache, body ache, anosmia, ageusia
			F, 34		60d	Mild diarrhea	Dry cough, diarrhea, tiredness, headache, body ache, anosmia, ageusia
Fonseca et al. [49]	Brazil	Case report	M, 29 (HCW)	–	225d	Fever, myalgia, cough, sore throat, and diarrhea	Fever, myalgia, cough, sore throat, and diarrhea
Nonaka et al. [50]	Brazil	Case report	F, 45 (HCW)	–	147d	Diarrhea, myalgia, asthenia, and onophagia	Headache, malaise, diarrhea, cough, and sore throat that evolved to myalgia and ageusia, muscle fatigue, insomnia, mild dyspnea on exertion, and shortness of breath
Ramírez et al. [51]	Colombia	Case report	F, 54	–	34d	Cough, fever, odynophagia and fatigue	Fever and odynophagia
Alshukairi et al. [52]	Saudi Arabia	Case report	F, 51	–	160d	fever, cough, malaise, and headache	progressive fever and dyspnea
Aguilar-Shea et al. [53]	Spain	Case report	M, 39 (HCW)	–	9m	Sore throat, fever, general malaise and nasal congestion, exertional tachycardia and chest pain anosmia and ageusia	Uncomfortable night sleep, sore throat on waking, slight general malaise, nasal congestion and nasal discharge
Mulder et al. [54]	Netherlands	Case report	F, 89	–	59d	Fever and severe cough	Fever, cough, and dyspnea
Dhar et al. [55]	India	Case report	M, 52	–	73d	Asymptomatic	Low-grade fever and body ache
Goldman et al. [56]	United States	Case report	–, 60–69(specific age unknown)	1/176	140d	Fever, chills, productive cough, dyspnea and chest pain	Dyspnea, dry cough and weakness(less)
Marquez et al. [57]	United States	Case report	F, 16	–	90d	Sore throat, fatigue, nasal congestion, rhinorrhea, and a nonproductive cough	Leg pain, swelling, fatigue, abdominal tenderness, fever
Buddingh et al. [58]	Netherlands	Case report	F, 16	–	13m	–	Mild respiratory symptoms
Tang et al. [59]	United States	Case report	F, 20s	–	19d	Cough, chills, exertional dyspnea, sore throat, dizziness, rhinorrhea, fever	Cough, fatigue, and dyspnea
Amorim et al. [60]	Brazil	Case report	F, 35 (HCW)	–	55d	Fever, headache, chills, sneezing, coryza, and myalgia	Headache, nasal congestion, odynophagia, ageusia, and anosmia
			F, 61 (HCW)		170d	Headache, cough, myalgia, dysphagy, coryza, diarrhea, and ageusia	Cough, myalgia, odynophagia, anosmia, and diarrhea
			F, 40 (HCW)		131d	Nasal congestion, coryza, cough, ageusia	Odynophagia, sneezing, coryza, diarrhea, ageusia, and anosmia
			F, 40 (HCW)		148d	Fever, headache, myalgia, coryza, dry cough, vomiting, and malaise	Odynophagia, dry cough, myalgia, malaise, coryza, and headache
Novazzi et al. [61]	Italy	Case report	M, 56	–	31d	Moderate dyspnea	–
			M, 58		30d	–	–
Salehi-Vaziri et al. [62]	Iran	Case report	M, 42	–	128d	Cough, headache and severe diarrhea	Body pain, shortness of breath, headache and anosmia
Romano et al. [63]	Brazil	Case report	F, 26	–	128d	Mild	Joint pain in the right leg, difficulty breathing, tiredness, dizziness and fatigue
Camargo et al. [64]	Brazil	Case report	F, 41	–	–	Headache, disseminated body pain, non-productive cough, shortness of breath, ageusia, and anosmia	Headache, cough, tiredness and myalgia
Brehm et al. [65]	Germany	Case report	F, 27 (HCW)	–	283d	Fever, chills, and exertional dyspnea	Dry cough and mild rhinorrhea
Tomkins-Tinch et al. [66]	United States	Case report	M, 61	–	111d	Fever, nausea, vomiting, and cough	Asymptomatic
Díaz et al. [67]	Panama	Case report	M, 36	–	181d	Myalgia, fever, cephalea, and rhinorrhea	Cephalea, myalgia and rhinorrhea
Yu et al. [68]	Brazil	Case report	F, 41 (HCW)	–	146d	Headache, myalgia, fatigue, fever, dry cough, dyspnea, anosmia ageusia	Headache, myalgia, fatigue, fever, dry cough, dyspnea, anosmia and ageusia, diarrhea, anorexia and dizziness
			F, 34 (HCW)		173d	Fever, cough, odynophagia and dyspnea	Headache, coryza, fever and sore throat
Zucman et al. [69]	France	Case report	M, 58	–	129d	Mild fever and dyspnea	Dyspnea and fever
Rani et al. [70]	India	Case report	M, 47	–	47d	Asymptomatic	Fever, cough, and malaise
Loconsole et al. [71]	Italy	Case report	F, 41 (HCW)	–	293d	Strong arthralgia, low-grade fever, headache, and diarrhea	Sore throat and headache
Selhorst et al. [72]	Belgium	Case report	F, 39 (HCW)	–	185d	Cough, dyspnea, headache, fever and general malaise	Milder
Van Elslande et al. [73]	Belgium	Case report	F, 51	–	3m	Headache, fever, myalgia, coughing, chest pain and dyspnea, anosmia and a change in taste	Headache, cough and fatigue
Jeffery-Smith et al. [74]	United Kingdom	Cross-sectional	–	–	–	Asymptomatic	–
Brouqui et al. [75]	France	Cross-sectional	25M, 21F 50 ± 22	46/6771	172d(90–308d)	Mild/moderate 37/39 (94.8); Severe/critical 2/39(5.1); Asymptomatic 7/46 (15.2);	Mild/moderate 26/33 (78.7); Severe/critical 7/33 (21.2); Asymptomatic 13/46 (28.2);
Abu-Raddad et al. [76]	Qatar	Cohort study	M, 35–39	–	–	Asymptomatic	–
			F, 40–44		–	Yes	–
			F, 35–39		–	Asymptomatic	–
			M, 35–39		–	Asymptomatic	–
			M, 30–34		–	Yes	–
dos Santos et al. [77]	Brazil	Case–control	M, 44 (HCW)	–	38d	Mild symptoms	Severe respiratory symptoms

Study	Reinfected patients (gender, age)	Lineage and Clade		Ct value		Infectivity	Co-morbidity	Vaccination	Outcome
		1st	2nd	1st	2nd				
**(b) Part 2**									
To et al. [8]	M, 33	GISAID clade V, B.2, 19A	GISAID clade G, B.1.79, 20A	–	26.69	–	None	–	–
Tillett et al. [9]	M, 25	20C	20C	–	–	–	None	–	Hospitalized
Borgogna et al. [10]	M, 52	B.1.1, 20B	B.1,20A	25–26(E, RdRp, and N)	34(E), 36(RNAseP), > 40 (RdRp)	–	Transitional cell carcinoma of the renal pelvis and ureter	–	Death
Gupta et al. [31]	M, 25 (HCW)	–	–	36	16.6	–	–	–	–
	F, 28 (HCW)	–	–	28.16	19.62	–	–	–	–
Larson et al. [32]	M, 42	B.1.26	B.1.26	–	–	–	–	–	–
Staub et al. [33]	M, 20s (HCW)	–	B1.351	–	–	–	–	–	–
	F, 20s (HCW)	–	B1.351	–	–	–	–	–	–
	M, 30s (HCW)	–	B1.351	–	–	–	–	–	–
	F, 20s (HCW)	–	B1.351	–	–	–	–	–	–
Salehi-Vaziri et al. [34]	F, 32	–	–	–	17(N),18(ORF1ab)	–	None	–	Recovery
	M, 54	–	–	27(N), 29(ORF1ab)	29(N), 30(ORF1ab)	–	None	–	Recovery
	M, 42	–	–	–	31(N), 33(ORF)	–	None	–	Recovery
Klein et al. [35]	M, 60–70 (specific age unknown	B.1	B.1.280	17.1(N1); 16.3(N2)	27.34(N1); 27.15(N2)	–	End-stage renal disease	–	Discharged
Shastri et al. [36]	M, 27 (HCW)	B, 20A	B.1.8, 19A	32(N); 32(ORF1ab)	25(N); 23(ORF1ab)	–	None	–	–
	F, 24 (HCW)	B.1, 19A	B.1.1.32, 20B	32(N); 34(ORF1ab)	17(N); 18(ORF1ab)	–	None	–	–
	F, 25 (HCW)	B.1.5, 19A	B.1, 20A	31(N); 31(ORF1ab)	22(N); 22(ORF1ab)	–	Hypertensive	–	–
Vetter et al. [37]	F, 36	20A	20A	–	–	–	–	–	–
Harrington et al. [38]	M, 78	B.2	B.1.1.7	26.8(E); 26.4(ORF1a)	27.5(E); 27.9(ORF1a)	–	Type 2 diabetes mellitus, diabetic nephropathy, COPD, mixed cenral and obstructive sleep apnea, ischemic heart disease	–	Hospitalized
Prado-Vivar et al. [39]	M, 46	B1.p9 20A	A.1.1 19B	36.85(ORF3a)	–	–	–	–	–
Moschetta et al. [40]	F, 39	–	Gamma VOC	–	–	–	–	CoronaVac COVID-19 vaccine	Recovered
	M, 49	–	Gamma VOC	–	–	–	–	first dose of the Astra-Zeneca COVID-19 vaccine	Recovered
Scarpati et al. [41]	M, 63(HCW)	20A	20E	–	–	–	Chronic obstructive pulmonary disease (COPD), type II diabetes, atrial fibrillation	first dose of Pfizer vaccination	Hospitalized
Massanella et al. [42]	M, 62 (HCW)	–	B.1.79	–	–	–	mild asthma, hypertension, dyslipidemia, liver steatosis, hyperuricemia, and overweight	–	Discharged
Garvey et al. [43]	M, 92	Sequencing failed	B.1.177	–	15.89	–	Dementia	–	Death
	M, 84	Sequencing failed	B.1.177	–	–	–	Dementia and Paget’s disease	–	–
	M, 59	Sequencing failed	B.1.1.7	–	–	–	End-stage renal failure	–	–
Siqueira et al. [44]	F, 76	–	–	34.21	11.99		Chronic renal failure and pyelonephritis	–	Death
Sevillano et al. [45]	M, 28	GISAID clade O, B.1.1 20B	GISAID clade O, B.1.1 20B	29.9	17.8	–	–	–	–
Kulkarni et al. [46]	M, 61	20B	20B	–	–	–	–	–	–
Lee et al. [47]	F, 21	V	G	23.11(E); 23.54(RdRP)	32.36 / 32.79 33.74 / 33.62	–	–	–	–
Fintelman-Rodrigues et al. [48]	M, 54	Not enough sample	20B	26.5	24.6	–	None	–	–
	M, 34	20B	20B	27.41	28.12	–	None	–	–
	F, 57	19A	20B	26.66	27.06	–	Discoid lupus erythematosus	–	–
	F, 34	Not enough sample	20B	28.48	24.5	–	None	–	–
Fonseca et al. [49]	M, 29 (HCW)	B.1.1.28	B.1.2	15.7(N1), 18.9(N2)	17.6(N1), 19.6(N2)	–	None	–	–
Nonaka et al. [50]	F, 45 (HCW)	B.1.1.33	B.1.1.28.2	25(N); 26(E); 27 (RdRp)	21(N); 12(E); 17(RdRp)	–	None	–	–
Ramírez et al. [51]	F, 54	B.1	B.1.1.269	21.2 (E); 24.5 (RdRp); 21.7 (N)	30.6 (E); 32.1(RdRp); 31.9 (N)	–	Hypertension, gastritis, and arthrosis	–	–
Alshukairi et al. [52]	F, 51	A	B.1.1.122	3	34	–	follicular non-Hodgkin lymphoma	One dose mRNA COVID-19 vaccine	Discharged
Aguilar-Shea et al. [53]	M, 39 (HCW)	–	B.1.1.7	–	–	–	None	–	Recovery
Mulder et al. [54]	F, 89	–	–	26.2(E)	25.2(E)	–	Waldenström macroglobulinemia	–	Death
Dhar et al. [55]	M, 52	B.1.0, 19A	B.1.36.1, 20A	36.04(ORF1ab); 36.74(E)	17.9(ORF1ab); 17.8(E)	–	–	–	–
Goldman et al. [56]	–, 60–69 (specific age unknown)	19B	20A	22.8 (E); 26.5 (RdRp)	43.3 (E); 39.6 (N2)	–	Severe emphysema	–	–
Marquez et al. [57]	F, 16	B.1.2	B.1.1.7	32.4(E) 32.0(S)	30.6(E) 31.0(S)	–	End-stage renal disease	–	–
Buddingh et al. [58]	F, 16	–	B.1.1.7	–	–	–	Multisystem inflammatory syndrome in children	–	Recovered
Tang et al. [59]	F, 20s	A.3	B.1.1	17.76	20.36	–	Asthma, obesity	–	–
Amorim et al. [60]	F, 35 (HCW)	B.1.1.33	B.1.1.28	35.24 (E); 40.12(N)	31.14(E); 31.3(N); 32.58(RdRp)	–	–	–	–
	F, 61 (HCW)	–	B.1.1.28	31.8(E)	20.45(E); 20.52(N); 22.65(RdRp)	–	–	–	–
	F, 40 (HCW)	–	–	35.15(E)	26.04(E); 26.88(N); 28.40(RdRp)	–	–	–	–
	F,40 (HCW)	–	B.1.1.28	34.80(E); 39.86(RdRp)	23.72(E); 23.48(N); 25.67(RdRp)	–	–	–	–
Novazzi et al. [61]	M, 56	Wuhan-Hu-1	B.1.1.7	–	–	–	Obesity and dyslipidemia	–	Hospitalized
	M,58	Wuhan-Hu-1	B.1.1.7	–	–	–	None	–	Hospitalized
Salehi-Vaziri et al. [62]	M, 42	20G	20G	18(N), 19(ORF1ab)	16(N),17(ORF1ab)	–	–	–	–
Romano et al. [63]	F, 26	Non-VOC virus	VOC virus P.1	21	24	–	Rheumatism	–	–
Camargo et al. [64]	F, 41	B.1.1.33	B.1.1.44	18(E),32(RNAseP)	22(E),30(RNAseP)		None	–	Discharged
Brehm et al. [65]	F, 27 (HCW)	B.3	B.1.177	–	–	–	None	–	–
Tomkins-Tinch et al. [66]	M, 61	–	–	–	–	–	Chronic hepatitis B and C	–	Discharged
Díaz et al. [67]	M, 36	A.2.4	GMI-PA584303	19(RdRp)	27(E), 28(RdRp).	–	None	–	Recovery
Yu et al. [68]	F, 41 (HCW)	B.1.1.33	B.1.1.28	–	–	–	None	–	–
	F, 34 (HCW)	B.1.1.28	P.2	–	–	–	Chronic respiratory disease	One dose	–
Zucman et al. [69]	M, 58	–	B.1.351	–	–	–	Asthma	–	–
Rani et al. [70]	M, 47	B.1.36	B.1.36	22.3(ORF1ab), 19.1(N)	21.9(ORF1ab), 19.2(N)	–	–	–	–
Loconsole et al. [71]	F, 41 (HCW)	B.1.1.74 GISAID clade GR, 20 B	B.1.177GISAID clade GV, 20 E	30(N);27(ORF1ab); 29(S)	15(N); 12(ORF1ab); 13(S)	–	None	One dose Comirnaty vaccine (Pfizer-BioNTech)	–
Selhorst et al. [72]	F, 39 (HCW)	V	G	Avg Ct 13	Avg Ct 19	–	–	–	–
Van Elslande et al. [73]	F, 51	B.1.1	A	25.6 (N1) 27.2 (N2)	32.6 (N1) 33.2 (N2)	–	Asthma	–	Recovery
Jeffery-Smith et al. [74]	–	–	B.1.36	–	–	–	–	–	–
Brouqui et al. [75]	25M, 21F 50 ± 22	–	–	–	–	–	None (20)	–	2 Death
Abu-Raddad et al. [76]	M, 35–39	–	–	–	–	–	–	–	–
	F, 40–44	–	–	–	22.2	–	–	–	–
	F, 35–39	–	–	–	–	–	–	–	–
	M, 35–39	–	–	–	–	–	–	–	–
	M, 30–34	–	–	–	–	–	–	–	–
Adrielle Dos Santos et al. [77]	M, 44 (HCW)	B.1	B.1.80	–	–	–	Obesity and systemic arterial hypertension	–	Death

HCW: Health Care Worker

^#^The words used to describe the symptoms in the table are from the original text

#### Symptoms of reinfected patients

Most reinfected patients show clinical symptoms, and only a few studies have reported patients being asymptomatic at both the first and secondary infections.

In the 36 studies (n = 51) [8–10, 32–37, 39, 43–45, 47–54, 56, 57, 59–62, 64–69, 71–73], which reported details of patients’ symptoms during the first infection, these commonly included cough (30, 62.3%), fever (31, 58.5%), headache (20, 37.7%), diarrhea (13, 24.5%), sore throat (12, 22.6%), myalgia (12, 22.6%), dyspnea (11, 20.8%), rhinitis (9, 17%), fatigue (7, 13.2%), chills (6, 11.3%), anosmia (5, 9.4%), ageusia (5, 9.4%), malaise (4, 7.5%), chest pain (4, 7.5%), nasal congestion (4, 7.5%), odynophagia (4, 7.5%), nausea (3 5.7%), vomiting (2, 3.8%), anxiety (2, 3.8%), lethargy (2, 3.8%), panic attacks (1, 1.9%), sneezing (1, 1.9%), confusion (1, 1.9%), body pain (1, 1.9%), arthralgia (1, 1.9%), exertional tachycardia (1, 1.9%), dizziness (1, 1.9%), and arthromyalgia (1, 1.9%), and 10 (18.9%) patients [31, 33, 36, 41, 46, 48, 55, 70] were asymptomatic.

At reinfection, 36 studies reported 54 patients [9, 32–39, 41, 43, 45–57, 59, 60, 62–65, 67–71] with common symptoms including cough (29, 51.8%), fever (26, 46.4%), headache (19, 33.9%), dyspnea (18, 32.1%), fatigue (17, 30.4%), myalgia (14, 25%), anosmia (10, 17.9%), diarrhea (8, 14.3%), sore throat (8, 14.3%), rhinitis(7, 12.5%), body pain(6, 10.7%), ageusia(6, 10.7%), odynophagia(6, 10.7%), malaise(4, 7.1%), nasal congestion (4, 7.1%), chill (3, 5.4%), dizziness (3, 5.4%), arthralgia (3, 5.4%), nausea (2, 3.6%), abdominal pain (2, 3.6%), anorexia (1, 1.8%), back pain (1, 1.8%), muscle fatigue (1, 1.8%), insomnia (1, 1.8%), hypoxia (1, 1.8%), gastrointestinal symptoms (1, 1.8%), leg pain (1, 1.8%), swelling (1, 1.8%), sneezing (1, 1.8%), lethargy (1, 1.8%), chest pain (1, 1.8%), shivering (1, 1.8%), respiratory failure (1, 1.8%),, and 9 (15.4%) patents [8, 31, 43, 66, 76] were asymptomatic.

#### Time from first to second clinical symptom

The shortest time from first infection to reinfection was 19 days [59] and the longest was 293 days [71].

#### Co-morbidity of reinfected patients

Thirty-four studies reported comorbidities in 64 patients [8–10, 34–36, 38, 41–44, 48–54, 56–59, 61, 63–69, 71, 73, 75, 77]. Among patients with co-morbidity, 10 had a combination of two or more chronic conditions [38, 41–44, 51, 59, 61, 66, 77]. Of these patients having comorbidities the youngest was 16 years old [58] and the oldest was 92 [43]. Hypertension and obesity were the most common comorbidities, followed by end-stage renal disease, asthma, chronic obstructive pulmonary disease (COPD), dementia, dyslipidemia and type 2 diabetes.

#### Vaccination

Two case reports reported on patients who had been vaccinated before reinfection. One patient developed reinfection 10 days after the first dose bur did not report the vaccine type [68]. Another patient developed reinfection 13 days after the first dose of Pfizer vaccination was administered [41].

#### Patient outcomes

Among the 21 studies that reported patient outcomes [9, 10, 34, 35, 38, 40–44, 52–54, 58, 61, 64, 66, 67, 73, 75, 77], nine patients (an age range from 16 to 54) recovered after reinfection [34, 40, 53, 58, 67, 73]. Seven patients died (aged 44–92): one died of septic shock and respiratory failure [10], another one died of respiratory failure [77], and the cause of death was not reported for the remaining five patients [43, 44, 54, 75]. Five patients were reported as still being hospitalized [38, 41, 61], and five patients had been discharged from hospital [35, 42, 52, 66].

#### Infectivity of reinfected patients

One case report showed that two days after diagnosis, one of the patient’s co-workers was also diagnosed with COVID-19 [63].

#### Treatment of first and second infections

At the first infection for the patients with reinfection, nine studies reported that 12 patients with COVID-19 were not treated [10, 38, 40, 47, 51, 52, 56, 60, 65]. Among the 9 studies reporting on 9 patients who had treatment [35, 41, 42, 48, 50, 53, 58, 61, 71], most patients received corticosteroids [61], including methylprednisolone [58], dexamethasone [41], and prednisone [50, 58]. Treatment with atazanavir and other antiviral drugs [35, 48], and tocilizumab [35, 41], and hydroxychloroquine was also common [35, 42]. Some patients also received levofloxacin [61], paracetamol [71], acetaminophen [53], and low molecular weight heparin [61]. And 4 patients were using a combination of drugs [35, 41, 58, 61].

For reinfected patients, 11 patients in 8 studies were untreated [8, 10, 35, 38, 40, 46, 51, 60]. Among the treated patients, most received prednisone [42, 61]and dexamethasone [42, 56, 69]. Treatment with remdesivir [42, 56], tocilizumab [42, 69], enoxaparin [42, 61], and azithromycin was also common [42, 61]. A few patients received inhaled salmeterol [42], amoxicillin-clavulanate [42] and convalescent plasma [66]. All of them were using combination drugs [42, 56, 61, 69].

#### Sequence analysis of reinfection cases

The B.1 variant strain was the most common one in the first infection. Variants B.1.1.7, B.1.128 and B.1.351 were the most common strains in reinfection. In the studies reporting the gene sequencing results in detail, 33 cases were infected by different strains [8, 10, 35, 36, 38, 39, 41, 47–49, 51, 52, 55–57, 59–61, 63–65, 67, 68, 71–73, 77]. Among them, the virus gene sequence of the first infection could not be detected in 2 cases, but epidemiological reports showed that the virus lineage of reinfection did not spread locally at the time of first infection [53, 58]. Eight patients were reported as being infected with the same strain (see Table 1) [9, 32, 37, 45, 46, 48, 62, 70].

#### Viral mutations of reinfected cases

In the included studies, viral gene sequencing revealed mutations among some patients. Of the 29 studies that reported mutations in details, D614G was the most common mutation [10, 34–36, 38, 39, 42, 47–49, 52, 60, 62, 64, 65, 67, 68, 70, 71], and other mutations such as N440K [70] and E484K [50, 68, 69] were also detected. See Additional file 1: Table S7.

## Discussion

We have systematically summarized and analyzed the characteristics of COVID-19 reinfected patients and the infecting viral gene sequences. In the current included studies, we found that reinfected patients usually have clinical symptoms. Reinfection events can occur within a short time, and there is a wide age distribution among reinfected patients. The B.1 variant strain was the most common one in the first infection, B.1.1.7, B.1.128 and B.1.351 variant strain were the most common strains in reinfection. And D614G was the most common mutation. Thirty-nine patients had no comorbidities and 10 had a combination of two or more chronic conditions. Nine patients (an age range from 16 to 54 years) recovered and 7 patients died after reinfection.

One cohort study reported that the incidence rate of reinfection was estimated at 0.66 per 10,000 person-weeks (95% CI: 0.56–0.78) [76]. Most reinfections constitute infection by different virus strains, but the virus gene sequencing of some patients showed that they were reinfected with the same strain as the first infection. Relevant animal experiments showed that after the second inoculation of the virus, no viral shedding from nasal, oropharyngeal, and rectal cavities was observed in these animals, and the virus was not transmitted to other animals [5, 6]. In our systematic review, there is only one study report of a patient infecting others. Thus, whether reinfected patients are infectious remains to be determined.

We think that reinfection is one of the reasons for re-detectable positive RNA test. Beyond that, the reason of patients with re-detectable positive RNA test including the results of Reverse Transcription-polymerase Chain Reaction (RT-PCR) may be a false negative at discharge or incomplete elimination of the virus [78]. The chief reasons for patients becoming reinfected are potentially as follows:

1. Insufficient immune capacity after the first infection. Individuals who recovered from COVID-19 have generally been thought to generate a robust immune response to clear the virus. Some studies have shown that the presence of SARS-CoV-2 antibodies confers subsequent immunity in most people for at least six to eight months [79, 80]. However, due to SARS-COV-2’s high variability, different genotypes and some human’s weak or non-lasting immune response, it remains to be determined whether the first infection confers protective immunity to subsequent infections.
2. Mutant viral strains. New virus variants such as B.1.1.7, P.1, and B.1.351 have emerged and become the main virus variants prevalent in many countries [12, 81, 82]. Some studies have indicated that P.1 has a 25–61% capacity to evade the immunity elicited by a previous infection caused by non-P.1 viruses [83]. The E484k mutation in these virus variants can, to a certain extent, escape recognition by people’s rehabilitation serum antibodies and make the virus variants have higher transmissibility [84, 85]. And the D614G mutation might help to increase the viral fitness in all emerging variants where this mutation is present. With the help of this mutation (D614G), the SARS-CoV-2 variants have gained viral fitness to enhance viral replication and increase transmission [86]. These S protein variants recently reported pose new potential challenges for the efficacy of vaccination, antibody-based therapies and viral diffusion control [87, 88].

With the continued emergence of variants of SARS-CoV-2, and the increased rate of disease transmission due to new variants, concerns have been raised about the practical effectiveness of vaccines [89]. Most COVID-19 vaccines elicit high levels of antibodies that target diverse regions of the spike protein, so some of the molecules are likely to be able to block variants of the virus [90]. One study found that the spike protein of the UK variant B.1.1.7 had little effect on sera from 16 subjects who received Pfizer vaccine injections [91]. By increasing the levels of cross-neutralizing antibodies, SARS-CoV-2 vaccination may strengthen protection, especially against variants harboring antibody escape mutations like B1.351 [92]. Protective immunity conferred by the mRNA vaccines is most likely to be retained against the B.1.617.1 and B.1.617.2 variants [93]. However, with the continuous mutation of the virus, the effectiveness of the vaccine for different variants remains to be studied.

Based on this study, we suggest that the management of reinfected patients should be consistent with the treatment of the first infection. These cases should be divided into mild, moderate and severe infection and given antiviral treatment. As a highly infectious virus, the modes of transmission include airborne, droplet, contact with contaminated surfaces, oral and fecal secretions [94]. With the emergence of new varieties, the transmission ability of new variants is increasing [95]. Thus, the public, including rehabilitated patients, should be fully vaccinated, wear masks in public places, and maintain social distance to avoid reinfection with the virus.

At the same time, our results found that the cause of death among patients who died was septic shock and respiratory failure. According to existing studies, lung disease is the most common long-term complication in patients with COVID-19 [96, 97], and the virus may also affect the cardiovascular system and nervous system [98]. Therefore, it is still necessary to conduct long-term follow-up studies to determine the various complications and prognosis of COVID-19 patients.

The current concept of reinfection is still not consistent. According to the European Centre for Disease Prevention and Control, reinfection is defined as “laboratory confirmation of two infections by two different strains (minimum distance to be determined or supported by phylogenetic and epidemiological data) with timely separated illness/infection episodes” [99]. The Centers for Disease Control and Prevention (CDC) uses the following criteria to define reinfection with SARS-CoV-2: detection of SARS-CoV-2 RNA (with Ct values < 33 if detected by RT-PCR) > 90 days after the first detection of viral RNA whether or not symptoms were present and paired respiratory specimens from each episode that belong to different clades of virus or have genomes with > 2 nucleotide differences per month [100]. The reinfection rate may vary greatly according to the different definitions of reinfection used. In screening the literature, we found that many studies, use RT-PCR positive as the standard for reinfection, but it has been stated that RT-PCR is meaningless when detecting reinfection as a positive RT-PCR test can only reflect the detection of RNA fragments that could be related to either a new viral infection, viral persistence with the reappearance of virus in mucosae, or viable viral debris [101]. Therefore, a positive RT-PCR test cannot be assumed to represent new viral infections in all situations.

Eight systematic reviews have already been published [20–27], but they have many limitations, such as not reporting the results of viral gene sequencing [20–22], or defining reinfection based on RT-PCR results [20, 27]. Thus, we decided to conduct this current review to address these limitations.

However, this current review also has some limitations. First, we only included data reported in the studies, and did not contact the authors for unreported data. Thus, we could not report the outcome measures concerned, such as the reinfection rate. In addition, the available evidence is still insufficient, and some relevant results, such as the infectivity of reinfected patients, the results of gene sequencing and vaccination, have not been reported. Second, In the cohort and cross-sectional studies, the possible factors for reinfection were not discussed. This also limits our discussion of factors posing a risk for reinfection. Third, for reports in which a patient was reinfected with the same strain, we relied on the report by the authors of the original study. But they did not report in detail how to distinguish between prolonged shedding of the virus and reinfection with the same strain. In addition, as patients with asymptomatic reinfections are usually found through the community testing for COVID-19 cases or Entry-exit screening of people at airport examinations, the number of reinfected persons may be seriously underestimated.

## Conclusions

In conclusion, our study shows that for some patients, the immune response to the first infection was not adequate to protect against reinfection. And reinfection is not specific to any specific strain. Therefore, individuals, regardless of history of prior infection, should continue to participate in mitigating the spread of infection by practicing social distancing and mask-wearing. More high-quality cohort studies based on viral gene sequencing are needed in the future to help us better understand the causes of reinfection and formulate vaccination strategies.

## Supplementary Information

** Additional file 1.** **Table S1.** Search strategy. **Table S2.** JBI assessment results of case reports. **Table S3.** JBI assessment results of cross-sectional studies. **Table S4.** JBI assessment results of case-control studies. **Table S5.** NOS assessment results of cohort studies. **Table S6.** Patients’ information. **Table S7.** Viral mutations of reinfection cases.

## Data Availability

The data used in this study were gathered from publicly available studies.

## Abbreviations

COVID-19: Coronavirus Disease 2019
SARS-CoV-2: Severe acute respiratory syndrome coronavirus 2
MERS-CoV: Middle East respiratory syndrome coronavirus
WHO: World Health Organization
VOIs: Variants of Interest
VOC: Variants of Concern
Ct: Cycle threshold
RT-PCR: Reverse Transcription-polymerase Chain Reaction
CDC: The Centers for Disease Control and Prevention
