# Supplementary material for: Reinfection in patients with COVID-19: a systematic review
Source: Glob Health Res Policy. 2022 Apr 29;7:12. doi: 10.1186/s41256-022-00245-3 (PMC9051013; doi:10.1186/s41256-022-00245-3)
Supplement: Supplementary file 1 — Additional file 1. Table S1. Search strategy. Table S2. JBI assessment results of case reports. Table S3. JBI assessment results of cross-sectional studies. Table S4. JBI assessment results of case-control studies. Table S5. NOS assessment results of cohort studies. Table S6. Patients’ information. Table S7. Viral mutations of reinfection cases. [file 41256_2022_245_MOESM1_ESM.docx]

**Supplementary Material**

| **Item** | **Page** |
| --- | --- |
| **Table S1. Search strategy** | **2-3** |
| **Table S2. JBI assessment results of case reports** | **4-24** |
| **Table S3. JBI assessment results of cross-sectional studies** | **24-25** |
| **Table S4. JBI assessment results of case-control studies** | **25** |
| **Table S5. NOS assessment results of cohort studies** | **25-26** |
| **Table S6. Patients’ information** | **26-30** |
| **Table S7. Viral mutations of reinfection cases** | **30-37** |

**Table S1. Search strategy**

Pubmed

#1 “SARS-CoV-2” [MeSH] OR “SARS-COV-2” [Title/Abstract] OR “Severe Acute Respiratory Syndrome Coronavirus 2” [Title/Abstract] OR “Coronavirus Disease 2019 Virus” [Title/Abstract] OR “2019 Novel Coronavirus” [Title/Abstract] OR “SARS-CoV-2 Virus” [Title/Abstract] OR “2019-nCoV” [Title/Abstract] OR “COVID-19 Virus” [Title/Abstract] OR “SARS Coronavirus 2” [Title/Abstract]

#2 “COVID-19” [MeSH] OR “COVID-19” [Title/Abstract] OR “COVID-19 Virus Disease” [Title/Abstract] OR “COVID19” [Title/Abstract] OR “COVID-19 Virus Infection” [Title/Abstract] OR “2019-nCoV Infection” [Title/Abstract] OR “Coronavirus Disease 2019” [Title/Abstract] OR “SARS Coronavirus 2 Infection” [Title/Abstract] OR “SARS CoV 2 Infection” [Title/Abstract] OR “COVID-19 Pandemic” [Title/Abstract]

#3 #1 OR #2

#4 “Reinfection” [MeSH] OR “Reinfection” [Title/Abstract] OR “Reactivated Infection” [Title/Abstract] OR “Recurrent Infection” [Title/Abstract] OR “secondary infection” [Title/Abstract] OR “second episode” [Title/Abstract]

#5 #3AND #4

Web of Science

#1 TI=(“SARS-COV-2” OR “Severe Acute Respiratory Syndrome Coronavirus 2” OR “Coronavirus Disease 2019 Virus” OR “2019 Novel Coronavirus” OR “SARS-CoV-2 Virus” OR “2019-nCoV” OR “COVID-19 Virus” OR “SARS Coronavirus 2”) OR AB=(“SARS-COV-2” OR “Severe Acute Respiratory Syndrome Coronavirus 2” OR “Coronavirus Disease 2019 Virus” OR “2019 Novel Coronavirus” OR “SARS-CoV-2 Virus” OR “2019-nCoV” OR “COVID-19 Virus” OR “SARS Coronavirus 2”)

#2 TI=(“COVID-19” OR “COVID-19 Virus Disease” OR “COVID19” OR “COVID-19 Virus Infection” OR “2019-nCoV Infection” OR “Coronavirus Disease 2019” OR “SARS Coronavirus 2 Infection” OR “SARS CoV 2 Infection” OR “COVID-19 Pandemic”) OR AB=(“COVID-19” OR “COVID-19 Virus Disease” OR “COVID19” OR “COVID-19 Virus Infection” OR “2019-nCoV Infection” OR “Coronavirus Disease 2019” OR “SARS Coronavirus 2 Infection” OR “SARS CoV 2 Infection” OR “COVID-19 Pandemic”)

#3 #1 OR #2

#4 TI=(“Reinfection” OR “Reactivated Infection” OR “Recurrent Infection” OR “secondary infection” OR “second episode”) OR AB=(“Reinfection” OR “Reactivated Infection” OR “Recurrent Infection” OR “secondary infection” OR “second episode”)

#5 #3AND #4

Embase

#1 'sars-cov-2'/exp OR 'sars-cov-2':ti,ab OR 'severe acute respiratory syndrome coronavirus 2':ti,ab OR 'coronavirus disease 2019 virus':ti,ab OR '2019 novel coronavirus':ti,ab OR 'sars-cov-2 virus':ti,ab OR '2019-ncov':ti,ab OR 'covid-19 virus':ti,ab OR 'sars coronavirus 2':ti,ab

#2 'covid 19'/exp OR 'covid 19':ti,ab OR 'covid-19 virus disease':ti,ab OR 'covid19':ti,ab OR 'covid-19 virus infection':ti,ab OR '2019-ncov infection':ti,ab OR 'coronavirus disease 2019':ti,ab OR 'sars coronavirus 2 infection':ti,ab OR 'sars cov 2 infection':ti,ab OR 'covid-19 pandemic':ti,ab

#3 #1 OR #2

#4 'Reinfection'/exp OR 'Reinfection':ti,ab OR 'Reactivated Infection':ti,ab OR 'Recurrent Infection':ti,ab OR 'secondary infection':ti,ab OR 'second episode':ti,ab

#5 #3AND #4

Cochrane

#1 MeSH descriptor: [SARS-COV-2] this term only

#2 (SARS-CoV-2):ti,ab,kw OR (2019 nCoV):ti,ab,kw OR (Severe Acute Respiratory Syndrome Coronavirus 2):ti,ab,kw OR (Coronavirus Disease 2019 Virus):ti,ab,kw OR (2019 Novel Coronavirus):ti,ab,kw

#3 (SARS-CoV-2 Virus):ti,ab,kw OR (COVID-19 Virus):ti,ab,kw OR (SARS Coronavirus 2):ti,ab,kw

#4 #1 OR #2 OR #3

#5 MeSH descriptor: [COVID-19] this term only

#6 (COVID-19):ti,ab,kw OR (COVID-19 Virus Disease):ti,ab,kw OR (COVID19):ti,ab,kw OR (COVID-19 Virus Infection):ti,ab,kw OR (2019-nCoV Infection):ti,ab,kw

#7 (Coronavirus Disease 2019):ti,ab,kw OR (SARS Coronavirus 2 Infection):ti,ab,kw OR (SARS CoV 2 Infection):ti,ab,kw OR (COVID-19 Pandemic):ti,ab,kw

#8 #5 OR #6 OR #7

#9 #4 OR #8

#10 MeSH descriptor: [COVID-19] this term only

#11 (Reinfection):ti,ab,kw OR (Reactivated Infection):ti,ab,kw OR (Reactivated Infection):ti,ab,kw OR (secondary infection):ti,ab,kw OR (second episode):ti,ab,kw

#12 #10 OR #11

#13 #9 AND #12

### Table S2. JBI assessment results of case reports

| **Study name: Ana Lucia Frugis Yu 2021/6/18** | |
| --- | --- |
| **Items** | **Response options** |
| *1.Were patient’s demographic characteristics clearly described?* | **Y** |
| *2.**Was the patient’s history clearly described and presented as a timeline?* | **Y** |
| *3.Was the current clinical condition of the patient on presentation clearly described?* | **Y** |
| *4.Were diagnostic tests or assessment methods and the results clearly described?* | **Y** |
| *5.Was the intervention(s) or treatment procedure(s) clearly described?* | **N** |
| *6.Was the post-intervention clinical condition clearly described?* | **N** |
| *7.Were adverse events (harms) or unanticipated events identified and described?* | **NA** |
| *8.Does the case report provide takeaway lessons?* | **Y** |

| **Study name: Antonio L. Aguilar-Shea 2021/5/7** | |
| --- | --- |
| **Items** | **Response options** |
| *1.Were patient’s demographic characteristics clearly described?* | **Y** |
| *2.Was the patient’s history clearly described and presented as a timeline?* | **Y** |
| *3.Was the current clinical condition of the patient on presentation clearly described?* | **Y** |
| *4.Were diagnostic tests or assessment methods and the results clearly described?* | **Y** |
| *5.Was the intervention(s) or treatment procedure(s) clearly described?* | **Y** |
| *6.Was the post-intervention clinical condition clearly described?* | **Y** |
| *7.Were adverse events (harms) or unanticipated events identified and described?* | **NA** |
| *8.Does the case report provide takeaway lessons?* | **Y** |

| **Study name: Abeer N. Alshukairi** 2021/7/18 | |
| --- | --- |
| **Items** | **Response options** |
| *1.Were patient’s demographic characteristics clearly described?* | **Y** |
| *2.Was the patient’s history clearly described and presented as a timeline?* | **Y** |
| *3.Was the current clinical condition of the patient on presentation clearly described?* | **Y** |
| *4.Were diagnostic tests or assessment methods and the results clearly described?* | **Y** |
| *5.Was the intervention(s) or treatment procedure(s) clearly described?* | **Y** |
| *6.Was the post-intervention clinical condition clearly described?* | **Y** |
| *7.Were adverse events (harms) or unanticipated events identified and described?* | **NA** |
| *8.Does the case report provide takeaway lessons?* | **Y** |

| **Study name: Belén Prado-Vivar** | |
| --- | --- |
| **Items** | **Response options** |
| *1.Were patient’s demographic characteristics clearly described?* | **Y** |
| *2.Was the patient’s history clearly described and presented as a timeline?* | **Y** |
| *3.Was the current clinical condition of the patient on presentation clearly described?* | **Y** |
| *4.Were diagnostic tests or assessment methods and the results clearly described?* | **Y** |
| *5.Was the intervention(s) or treatment procedure(s) clearly described?* | **N** |
| *6.Was the post-intervention clinical condition clearly described?* | **N** |
| *7.Were adverse events (harms) or unanticipated events identified and described?* | **NA** |
| *8.Does the case report provide takeaway lessons?* | **Y** |

| **Study name: Camila Malta Romano 2021/4/23** | |
| --- | --- |
| **Items** | **Response options** |
| *1.Were patient’s demographic characteristics clearly described?* | **Y** |
| *2.Was the patient’s history clearly described and presented as a timeline?* | **Y** |
| *3.Was the current clinical condition of the patient on presentation clearly described?* | **Y** |
| *4.Were diagnostic tests or assessment methods and the results clearly described?* | **Y** |
| *5.Was the intervention(s) or treatment procedure(s) clearly described?* | **N** |
| *6.Was the post-intervention clinical condition clearly described?* | **N** |
| *7.Were adverse events (harms) or unanticipated events identified and described?* | **NA** |
| *8.Does the case report provide takeaway lessons?* | **Y** |

| **Study name: Carolina Kymie Vasques Nonaka 2021/5/27** | |
| --- | --- |
| **Items** | **Response options** |
| *1.Were patient’s demographic characteristics clearly described?* | **Y** |
| *2.Was the patient’s history clearly described and presented as a timeline?* | **Y** |
| *3.Was the current clinical condition of the patient on presentation clearly described?* | **Y** |
| *4.Were diagnostic tests or assessment methods and the results clearly described?* | **Y** |
| *5.Was the intervention(s) or treatment procedure(s) clearly described?* | **Y** |
| *6.Was the post-intervention clinical condition clearly described?* | **N** |
| *7.Were adverse events (harms) or unanticipated events identified and described?* | **NA** |
| *8.Does the case report provide takeaway lessons?* | **Y** |

| **Study name: Christopher H. Tomkins-Tinch 2021/4/20** | |
| --- | --- |
| **Items** | **Response options** |
| *1.Were patient’s demographic characteristics clearly described?* | **Y** |
| *2.Was the patient’s history clearly described and presented as a timeline?* | **Y** |
| *3.Was the current clinical condition of the patient on presentation clearly described?* | **Y** |
| *4.Were diagnostic tests or assessment methods and the results clearly described?* | **Y** |
| *5.Was the intervention(s) or treatment procedure(s) clearly described?* | **Y** |
| *6.Was the post-intervention clinical condition clearly described?* | **N** |
| *7.Were adverse events (harms) or unanticipated events identified and described?* | **NA** |
| *8.Does the case report provide takeaway lessons?* | **Y** |

| **Study name: Cynthia Y. Tang 2021/6/25** | |
| --- | --- |
| **Items** | **Response options** |
| *1.Were patient’s demographic characteristics clearly described?* | **Y** |
| *2.Was the patient’s history clearly described and presented as a timeline?* | **Y** |
| *3.Was the current clinical condition of the patient on presentation clearly described?* | **Y** |
| *4.Were diagnostic tests or assessment methods and the results clearly described?* | **Y** |
| *5.Was the intervention(s) or treatment procedure(s) clearly described?* | **N** |
| *6.Was the post-intervention clinical condition clearly described?* | **N** |
| *7.Were adverse events (harms) or unanticipated events identified and described?* | **NA** |
| *8.Does the case report provide takeaway lessons?* | **Y** |

| **Study name: Carlos Henrique Camargo 2021/8/20** | |
| --- | --- |
| **Items** | **Response options** |
| *1.Were patient’s demographic characteristics clearly described?* | **Y** |
| *2.Was the patient’s history clearly described and presented as a timeline?* | **Y** |
| *3.Was the current clinical condition of the patient on presentation clearly described?* | **Y** |
| *4.Were diagnostic tests or assessment methods and the results clearly described?* | **Y** |
| *5.Was the intervention(s) or treatment procedure(s) clearly described?* | **Y** |
| *6.Was the post-intervention clinical condition clearly described?* | **N** |
| *7.Were adverse events (harms) or unanticipated events identified and described?* | **NA** |
| *8.Does the case report provide takeaway lessons?* | **Y** |

| **Study name: Cinzia Borgogna 2020/7/14** | |
| --- | --- |
| **Items** | **Response options** |
| *1.Were patient’s demographic characteristics clearly described?* | **Y** |
| *2.Was the patient’s history clearly described and presented as a timeline?* | **Y** |
| *3.Was the current clinical condition of the patient on presentation clearly described?* | **Y** |
| *4.Were diagnostic tests or assessment methods and the results clearly described?* | **Y** |
| *5.Was the intervention(s) or treatment procedure(s) clearly described?* | **Y** |
| *6.Was the post-intervention clinical condition clearly described?* | **Y** |
| *7.Were adverse events (harms) or unanticipated events identified and described?* | **NA** |
| *8.Does the case report provide takeaway lessons?* | **Y** |

| **Study name: Derek Larson 2020/9/19** | |
| --- | --- |
| **Items** | **Response options** |
| *1.Were patient’s demographic characteristics clearly described?* | **Y** |
| *2.Was the patient’s history clearly described and presented as a timeline?* | **Y** |
| *3.Was the current clinical condition of the patient on presentation clearly described?* | **Y** |
| *4.Were diagnostic tests or assessment methods and the results clearly described?* | **Y** |
| *5.Was the intervention(s) or treatment procedure(s) clearly described?* | **N** |
| *6.Was the post-intervention clinical condition clearly described?* | **N** |
| *7.Were adverse events (harms) or unanticipated events identified and described?* | **NA** |
| *8.Does the case report provide takeaway lessons?* | **N** |

| **Study name: Daniela Loconsole 2021/5/12** | |
| --- | --- |
| **Items** | **Response options** |
| *1.Were patient’s demographic characteristics clearly described?* | **Y** |
| *2.Was the patient’s history clearly described and presented as a timeline?* | **Y** |
| *3.Was the current clinical condition of the patient on presentation clearly described?* | **Y** |
| *4.Were diagnostic tests or assessment methods and the results clearly described?* | **Y** |
| *5.Was the intervention(s) or treatment procedure(s) clearly described?* | **Y** |
| *6.Was the post-intervention clinical condition clearly described?* | **Y** |
| *7.Were adverse events (harms) or unanticipated events identified and described?* | **NA** |
| *8.Does the case report provide takeaway lessons?* | **Y** |

| **Study name: David Harrington 2021/1/9** | |
| --- | --- |
| **Items** | **Response options** |
| *1.Were patient’s demographic characteristics clearly described?* | **Y** |
| *2.Was the patient’s history clearly described and presented as a timeline?* | **Y** |
| *3.Was the current clinical condition of the patient on presentation clearly described?* | **Y** |
| *4.Were diagnostic tests or assessment methods and the results clearly described?* | **Y** |
| *5.Was the intervention(s) or treatment procedure(s) clearly described?* | **Y** |
| *6.Was the post-intervention clinical condition clearly described?* | **N** |
| *7.Were adverse events (harms) or unanticipated events identified and described?* | **NA** |
| *8.Does the case report provide takeaway lessons?* | **Y** |

| **Study name: Emilie P. Buddingh 2021/8/10** | |
| --- | --- |
| **Items** | **Response options** |
| *1.Were patient’s demographic characteristics clearly described?* | **Y** |
| *2.Was the patient’s history clearly described and presented as a timeline?* | **Y** |
| *3.Was the current clinical condition of the patient on presentation clearly described?* | **Y** |
| *4.Were diagnostic tests or assessment methods and the results clearly described?* | **Y** |
| *5.Was the intervention(s) or treatment procedure(s) clearly described?* | **Y** |
| *6.Was the post-intervention clinical condition clearly described?* | **Y** |
| *7.Were adverse events (harms) or unanticipated events identified and described?* | **NA** |
| *8.Does the case report provide takeaway lessons?* | **Y** |

| **Study name: Federica Novazzi 2021/5/10** | |
| --- | --- |
| **Items** | **Response options** |
| *1.Were patient’s demographic characteristics clearly described?* | **Y** |
| *2.Was the patient’s history clearly described and presented as a timeline?* | **Y** |
| *3.Was the current clinical condition of the patient on presentation clearly described?* | **Y** |
| *4.Were diagnostic tests or assessment methods and the results clearly described?* | **Y** |
| *5.Was the intervention(s) or treatment procedure(s) clearly described?* | **Y** |
| *6.Was the post-intervention clinical condition clearly described?* | **Y** |
| *7.Were adverse events (harms) or unanticipated events identified and described?* | **NA** |
| *8.Does the case report provide takeaway lessons?* | **Y** |

| **Study name: Gabriela Sevillano 2021/4/27** | |
| --- | --- |
| **Items** | **Response options** |
| *1.Were patient’s demographic characteristics clearly described?* | **Y** |
| *2.Was the patient’s history clearly described and presented as a timeline?* | **Y** |
| *3.Was the current clinical condition of the patient on presentation clearly described?* | **Y** |
| *4.Were diagnostic tests or assessment methods and the results clearly described?* | **Y** |
| *5.Was the intervention(s) or treatment procedure(s) clearly described?* | **N** |
| *6.Was the post-intervention clinical condition clearly described?* | **N** |
| *7.Were adverse events (harms) or unanticipated events identified and described?* | **NA** |
| *8.Does the case report provide takeaway lessons?* | **Y** |

| **Study name: Giuliana Scarpati 2020/7/13** | |
| --- | --- |
| **Items** | **Response options** |
| *1.Were patient’s demographic characteristics clearly described?* | **Y** |
| *2.Was the patient’s history clearly described and presented as a timeline?* | **Y** |
| *3.Was the current clinical condition of the patient on presentation clearly described?* | **Y** |
| *4.Were diagnostic tests or assessment methods and the results clearly described?* | **Y** |
| *5.Was the intervention(s) or treatment procedure(s) clearly described?* | **Y** |
| *6.Was the post-intervention clinical condition clearly described?* | **Y** |
| *7.Were adverse events (harms) or unanticipated events identified and described?* | **NA** |
| *8.Does the case report provide takeaway lessons?* | **Y** |

| **Study name: Jan Van Elslande 2020/9/5** | |
| --- | --- |
| **Items** | **Response options** |
| *1.Were patient’s demographic characteristics clearly described?* | **Y** |
| *2.Was the patient’s history clearly described and presented as a timeline?* | **Y** |
| *3.Was the current clinical condition of the patient on presentation clearly described?* | **Y** |
| *4.Were diagnostic tests or assessment methods and the results clearly described?* | **Y** |
| *5.Was the intervention(s) or treatment procedure(s) clearly described?* | **N** |
| *6.Was the post-intervention clinical condition clearly described?* | **N** |
| *7.Were adverse events (harms) or unanticipated events identified and described?* | **NA** |
| *8.Does the case report provide takeaway lessons?* | **Y** |

| **Study name: Jason D. Goldman 2020/9/25** | |
| --- | --- |
| **Items** | **Response options** |
| *1.Were patient’s demographic characteristics clearly described?* | **N** |
| *2.Was the patient’s history clearly described and presented as a timeline?* | **Y** |
| *3.Was the current clinical condition of the patient on presentation clearly described?* | **Y** |
| *4.Were diagnostic tests or assessment methods and the results clearly described?* | **Y** |
| *5.Was the intervention(s) or treatment procedure(s) clearly described?* | **N** |
| *6.Was the post-intervention clinical condition clearly described?* | **N** |
| *7.Were adverse events (harms) or unanticipated events identified and described?* | **NA** |
| *8.Does the case report provide takeaway lessons?* | **Y** |

| **Study name: Jayanthi Shastri 2021/3/9** | |
| --- | --- |
| **Items** | **Response options** |
| *1.Were patient’s demographic characteristics clearly described?* | **Y** |
| *2.Was the patient’s history clearly described and presented as a timeline?* | **Y** |
| *3.Was the current clinical condition of the patient on presentation clearly described?* | **Y** |
| *4.Were diagnostic tests or assessment methods and the results clearly described?* | **Y** |
| *5.Was the intervention(s) or treatment procedure(s) clearly described?* | **N** |
| *6.Was the post-intervention clinical condition clearly described?* | **N** |
| *7.Were adverse events (harms) or unanticipated events identified and described?* | **NA** |
| *8.Does the case report provide takeaway lessons?* | **Y** |

| **Study name: Jee-Soo Lee 2020/11/21** | |
| --- | --- |
| **Items** | **Response options** |
| *1.Were patient’s demographic characteristics clearly described?* | **Y** |
| *2.Was the patient’s history clearly described and presented as a timeline?* | **Y** |
| *3.Was the current clinical condition of the patient on presentation clearly described?* | **Y** |
| *4.Were diagnostic tests or assessment methods and the results clearly described?* | **Y** |
| *5.Was the intervention(s) or treatment procedure(s) clearly described?* | **Y** |
| *6.Was the post-intervention clinical condition clearly described?* | **Y** |
| *7.Were adverse events (harms) or unanticipated events identified and described?* | **NA** |
| *8.Does the case report provide takeaway lessons?* | **Y** |

| **Study name: Jonathan Klein 2021/3/26** | |
| --- | --- |
| **Items** | **Response options** |
| *1.Were patient’s demographic characteristics clearly described?* | **Y** |
| *2.Was the patient’s history clearly described and presented as a timeline?* | **Y** |
| *3.Was the current clinical condition of the patient on presentation clearly described?* | **Y** |
| *4.Were diagnostic tests or assessment methods and the results clearly described?* | **Y** |
| *5.Was the intervention(s) or treatment procedure(s) clearly described?* | **Y** |
| *6.Was the post-intervention clinical condition clearly described?* | **Y** |
| *7.Were adverse events (harms) or unanticipated events identified and described?* | **NA** |
| *8.Does the case report provide takeaway lessons?* | **Y** |

| **Study name: Juan David Ramírez 2021/3/19** | |
| --- | --- |
| **Items** | **Response options** |
| *1.Were patient’s demographic characteristics clearly described?* | **Y** |
| *2.Was the patient’s history clearly described and presented as a timeline?* | **Y** |
| *3.Was the current clinical condition of the patient on presentation clearly described?* | **Y** |
| *4.Were diagnostic tests or assessment methods and the results clearly described?* | **Y** |
| *5.Was the intervention(s) or treatment procedure(s) clearly described?* | **N** |
| *6.Was the post-intervention clinical condition clearly described?* | **N** |
| *7.Were adverse events (harms) or unanticipated events identified and described?* | **NA** |
| *8.Does the case report provide takeaway lessons?* | **Y** |

| **Study name: Juliana D. Siqueira 2020/6** | |
| --- | --- |
| **Items** | **Response options** |
| *1.Were patient’s demographic characteristics clearly described?* | **Y** |
| *2.Was the patient’s history clearly described and presented as a timeline?* | **Y** |
| *3.Was the current clinical condition of the patient on presentation clearly described?* | **Y** |
| *4.Were diagnostic tests or assessment methods and the results clearly described?* | **Y** |
| *5.Was the intervention(s) or treatment procedure(s) clearly described?* | **N** |
| *6.Was the post-intervention clinical condition clearly described?* | **N** |
| *7.Were adverse events (harms) or unanticipated events identified and described?* | **NA** |
| *8.Does the case report provide takeaway lessons?* | **Y** |

| **Study name: Kelvin Kai-Wang To 2020/8/25** | |
| --- | --- |
| **Items** | **Response options** |
| *1.Were patient’s demographic characteristics clearly described?* | **Y** |
| *2.Was the patient’s history clearly described and presented as a timeline?* | **Y** |
| *3.Was the current clinical condition of the patient on presentation clearly described?* | **Y** |
| *4.Were diagnostic tests or assessment methods and the results clearly described?* | **Y** |
| *5.Was the intervention(s) or treatment procedure(s) clearly described?* | **N** |
| *6.Was the post-intervention clinical condition clearly described?* | **N** |
| *7.Were adverse events (harms) or unanticipated events identified and described?* | **NA** |
| *8.Does the case report provide takeaway lessons?* | **Y** |

| **Study name: Lucila Marquez 2021/1/1** | |
| --- | --- |
| **Items** | **Response options** |
| *1.Were patient’s demographic characteristics clearly described?* | **Y** |
| *2.Was the patient’s history clearly described and presented as a timeline?* | **Y** |
| *3.Was the current clinical condition of the patient on presentation clearly described?* | **Y** |
| *4.Were diagnostic tests or assessment methods and the results clearly described?* | **Y** |
| *5.Was the intervention(s) or treatment procedure(s) clearly described?* | **N** |
| *6.Was the post-intervention clinical condition clearly described?* | **N** |
| *7.Were adverse events (harms) or unanticipated events identified and described?* | **NA** |
| *8.Does the case report provide takeaway lessons?* | **Y** |

| **Study name: Marina Oliboni Moschetta 2021/8/12** | |
| --- | --- |
| **Items** | **Response options** |
| *1.Were patient’s demographic characteristics clearly described?* | **Y** |
| *2.Was the patient’s history clearly described and presented as a timeline?* | **Y** |
| *3.Was the current clinical condition of the patient on presentation clearly described?* | **Y** |
| *4.Were diagnostic tests or assessment methods and the results clearly described?* | **Y** |
| *5.Was the intervention(s) or treatment procedure(s) clearly described?* | **N** |
| *6.Was the post-intervention clinical condition clearly described?* | **N** |
| *7.Were adverse events (harms) or unanticipated events identified and described?* | **NA** |
| *8.Does the case report provide takeaway lessons?* | **Y** |

| **Study name: Marta Massanella 2021/6/23** | |
| --- | --- |
| **Items** | **Response options** |
| *1.Were patient’s demographic characteristics clearly described?* | **Y** |
| *2.Was the patient’s history clearly described and presented as a timeline?* | **Y** |
| *3.Was the current clinical condition of the patient on presentation clearly described?* | **Y** |
| *4.Were diagnostic tests or assessment methods and the results clearly described?* | **Y** |
| *5.Was the intervention(s) or treatment procedure(s) clearly described?* | **Y** |
| *6.Was the post-intervention clinical condition clearly described?* | **Y** |
| *7.Were adverse events (harms) or unanticipated events identified and described?* | **NA** |
| *8.Does the case report provide takeaway lessons?* | **Y** |

| **Study name: Mahesh S. Dhar 2021/4/12** | |
| --- | --- |
| **Items** | **Response options** |
| *1.Were patient’s demographic characteristics clearly described?* | **Y** |
| *2.Was the patient’s history clearly described and presented as a timeline?* | **Y** |
| *3.Was the current clinical condition of the patient on presentation clearly described?* | **Y** |
| *4.Were diagnostic tests or assessment methods and the results clearly described?* | **Y** |
| *5.Was the intervention(s) or treatment procedure(s) clearly described?* | **N** |
| *6.Was the post-intervention clinical condition clearly described?* | **N** |
| *7.Were adverse events (harms) or unanticipated events identified and described?* | **NA** |
| *8.Does the case report provide takeaway lessons?* | **Y** |

| **Study name: Mariene R. Amorim 2021/1** | |
| --- | --- |
| **Items** | **Response options** |
| *1.Were patient’s demographic characteristics clearly described?* | **Y** |
| *2.Was the patient’s history clearly described and presented as a timeline?* | **Y** |
| *3.Was the current clinical condition of the patient on presentation clearly described?* | **Y** |
| *4.Were diagnostic tests or assessment methods and the results clearly described?* | **Y** |
| *5.Was the intervention(s) or treatment procedure(s) clearly described?* | **N** |
| *6.Was the post-intervention clinical condition clearly described?* | **N** |
| *7.Were adverse events (harms) or unanticipated events identified and described?* | **NA** |
| *8.Does the case report provide takeaway lessons?* | **Y** |

| **Study name: Mark I. Garvey 2021/1** | |
| --- | --- |
| **Items** | **Response options** |
| *1.Were patient’s demographic characteristics clearly described?* | **Y** |
| *2.Was the patient’s history clearly described and presented as a timeline?* | **Y** |
| *3.Was the current clinical condition of the patient on presentation clearly described?* | **Y** |
| *4.Were diagnostic tests or assessment methods and the results clearly described?* | **Y** |
| *5.Was the intervention(s) or treatment procedure(s) clearly described?* | **N** |
| *6.Was the post-intervention clinical condition clearly described?* | **N** |
| *7.Were adverse events (harms) or unanticipated events identified and described?* | **NA** |
| *8.Does the case report provide takeaway lessons?* | **Y** |

| **Study name: Marlies Mulder 2020/10/9** | |
| --- | --- |
| **Items** | **Response options** |
| *1.Were patient’s demographic characteristics clearly described?* | **Y** |
| *2.Was the patient’s history clearly described and presented as a timeline?* | **Y** |
| *3.Was the current clinical condition of the patient on presentation clearly described?* | **Y** |
| *4.Were diagnostic tests or assessment methods and the results clearly described?* | **Y** |
| *5.Was the intervention(s) or treatment procedure(s) clearly described?* | **N** |
| *6.Was the post-intervention clinical condition clearly described?* | **N** |
| *7.Were adverse events (harms) or unanticipated events identified and described?* | **NA** |
| *8.Does the case report provide takeaway lessons?* | **Y** |

| **Study name: Mostafa Salehi-Vaziri(3) 2021/1/2** | |
| --- | --- |
| **Items** | **Response options** |
| *1.Were patient’s demographic characteristics clearly described?* | **Y** |
| *2.Was the patient’s history clearly described and presented as a timeline?* | **Y** |
| *3.Was the current clinical condition of the patient on presentation clearly described?* | **Y** |
| *4.Were diagnostic tests or assessment methods and the results clearly described?* | **Y** |
| *5.Was the intervention(s) or treatment procedure(s) clearly described?* | **N** |
| *6.Was the post-intervention clinical condition clearly described?* | **N** |
| *7.Were adverse events (harms) or unanticipated events identified and described?* | **NA** |
| *8.Does the case report provide takeaway lessons?* | **Y** |

| **Study name: Mostafa Salehi-Vaziri 2021/4/1** | |
| --- | --- |
| **Items** | **Response options** |
| *1.Were patient’s demographic characteristics clearly described?* | **Y** |
| *2.Was the patient’s history clearly described and presented as a timeline?* | **Y** |
| *3.Was the current clinical condition of the patient on presentation clearly described?* | **Y** |
| *4.Were diagnostic tests or assessment methods and the results clearly described?* | **Y** |
| *5.Was the intervention(s) or treatment procedure(s) clearly described?* | **N** |
| *6.Was the post-intervention clinical condition clearly described?* | **N** |
| *7.Were adverse events (harms) or unanticipated events identified and described?* | **NA** |
| *8.Does the case report provide takeaway lessons?* | **Y** |

| **Study name: Natalia Fintelman-Rodrigues 2021/5** | |
| --- | --- |
| **Items** | **Response options** |
| *1.Were patient’s demographic characteristics clearly described?* | **Y** |
| *2.Was the patient’s history clearly described and presented as a timeline?* | **Y** |
| *3.Was the current clinical condition of the patient on presentation clearly described?* | **Y** |
| *4.Were diagnostic tests or assessment methods and the results clearly described?* | **Y** |
| *5.Was the intervention(s) or treatment procedure(s) clearly described?* | **N** |
| *6.Was the post-intervention clinical condition clearly described?* | **N** |
| *7.Were adverse events (harms) or unanticipated events identified and described?* | **NA** |
| *8.Does the case report provide takeaway lessons?* | **Y** |

| **Study name: Noémie Zucman 2021/2/10** | |
| --- | --- |
| **Items** | **Response options** |
| *1.Were patient’s demographic characteristics clearly described?* | **Y** |
| *2.Was the patient’s history clearly described and presented as a timeline?* | **Y** |
| *3.Was the current clinical condition of the patient on presentation clearly described?* | **Y** |
| *4.Were diagnostic tests or assessment methods and the results clearly described?* | **Y** |
| *5.Was the intervention(s) or treatment procedure(s) clearly described?* | **Y** |
| *6.Was the post-intervention clinical condition clearly described?* | **Y** |
| *7.Were adverse events (harms) or unanticipated events identified and described?* | **NA** |
| *8.Does the case report provide takeaway lessons?* | **Y** |

| **Study name: Onkar Kulkarn 2021/2/16** | |
| --- | --- |
| **Items** | **Response options** |
| *1.Were patient’s demographic characteristics clearly described?* | **Y** |
| *2.Was the patient’s history clearly described and presented as a timeline?* | **Y** |
| *3.Was the current clinical condition of the patient on presentation clearly described?* | **Y** |
| *4.Were diagnostic tests or assessment methods and the results clearly described?* | **Y** |
| *5.Was the intervention(s) or treatment procedure(s) clearly described?* | **N** |
| *6.Was the post-intervention clinical condition clearly described?* | **N** |
| *7.Were adverse events (harms) or unanticipated events identified and described?* | **NA** |
| *8.Does the case report provide takeaway lessons?* | **Y** |

| **Study name: Pallavali R. Rani 2021/07** | |
| --- | --- |
| **Items** | **Response options** |
| *1.Were patient’s demographic characteristics clearly described?* | **Y** |
| *2.Was the patient’s history clearly described and presented as a timeline?* | **Y** |
| *3.Was the current clinical condition of the patient on presentation clearly described?* | **Y** |
| *4.Were diagnostic tests or assessment methods and the results clearly described?* | **Y** |
| *5.Was the intervention(s) or treatment procedure(s) clearly described?* | **N** |
| *6.Was the post-intervention clinical condition clearly described?* | **N** |
| *7.Were adverse events (harms) or unanticipated events identified and described?* | **NA** |
| *8.Does the case report provide takeaway lessons?* | **Y** |

| **Study name: Pauline Vetter 2021/2/20** | |
| --- | --- |
| **Items** | **Response options** |
| *1.Were patient’s demographic characteristics clearly described?* | **Y** |
| *2.Was the patient’s history clearly described and presented as a timeline?* | **Y** |
| *3.Was the current clinical condition of the patient on presentation clearly described?* | **Y** |
| *4.Were diagnostic tests or assessment methods and the results clearly described?* | **Y** |
| *5.Was the intervention(s) or treatment procedure(s) clearly described?* | **N** |
| *6.Was the post-intervention clinical condition clearly described?* | **N** |
| *7.Were adverse events (harms) or unanticipated events identified and described?* | **NA** |
| *8.Does the case report provide takeaway lessons?* | **Y** |

| **Study name: Philippe Selhorst 2020/12/14** | |
| --- | --- |
| **Items** | **Response options** |
| *1.Were patient’s demographic characteristics clearly described?* | **Y** |
| *2.Was the patient’s history clearly described and presented as a timeline?* | **Y** |
| *3.Was the current clinical condition of the patient on presentation clearly described?* | **Y** |
| *4.Were diagnostic tests or assessment methods and the results clearly described?* | **Y** |
| *5.Was the intervention(s) or treatment procedure(s) clearly described?* | **N** |
| *6.Was the post-intervention clinical condition clearly described?* | **N** |
| *7.Were adverse events (harms) or unanticipated events identified and described?* | **NA** |
| *8.Does the case report provide takeaway lessons?* | **Y** |

| **Study name: Richard L Tillett 2020/10/12** | |
| --- | --- |
| **Items** | **Response options** |
| *1.Were patient’s demographic characteristics clearly described?* | **Y** |
| *2.Was the patient’s history clearly described and presented as a timeline?* | **Y** |
| *3.Was the current clinical condition of the patient on presentation clearly described?* | **Y** |
| *4.Were diagnostic tests or assessment methods and the results clearly described?* | **Y** |
| *5.Was the intervention(s) or treatment procedure(s) clearly described?* | **N** |
| *6.Was the post-intervention clinical condition clearly described?* | **N** |
| *7.Were adverse events (harms) or unanticipated events identified and described?* | **NA** |
| *8.Does the case report provide takeaway lessons?* | **Y** |

| **Study name: Thérèse Staub 2021/04/06** | |
| --- | --- |
| **Items** | **Response options** |
| *1.Were patient’s demographic characteristics clearly described?* | **Y** |
| *2.Was the patient’s history clearly described and presented as a timeline?* | **Y** |
| *3.Was the current clinical condition of the patient on presentation clearly described?* | **Y** |
| *4.Were diagnostic tests or assessment methods and the results clearly described?* | **Y** |
| *5.Was the intervention(s) or treatment procedure(s) clearly described?* | **N** |
| *6.Was the post-intervention clinical condition clearly described?* | **N** |
| *7.Were adverse events (harms) or unanticipated events identified and described?* | **NA** |
| *8.Does the case report provide takeaway lessons?* | **Y** |

| **Study name: Thomas Theo Brehm 2021/04/12** | |
| --- | --- |
| **Items** | **Response options** |
| *1.Were patient’s demographic characteristics clearly described?* | **Y** |
| *2.Was the patient’s history clearly described and presented as a timeline?* | **Y** |
| *3.Was the current clinical condition of the patient on presentation clearly described?* | **Y** |
| *4.Were diagnostic tests or assessment methods and the results clearly described?* | **Y** |
| *5.Was the intervention(s) or treatment procedure(s) clearly described?* | **Y** |
| *6.Was the post-intervention clinical condition clearly described?* | **Y** |
| *7.Were adverse events (harms) or unanticipated events identified and described?* | **NA** |
| *8.Does the case report provide takeaway lessons?* | **Y** |

| **Study name: Vivek Gupta 2020/09/23** | |
| --- | --- |
| **Items** | **Response options** |
| *1.Were patient’s demographic characteristics clearly described?* | **Y** |
| *2.Was the patient’s history clearly described and presented as a timeline?* | **Y** |
| *3.Was the current clinical condition of the patient on presentation clearly described?* | **Y** |
| *4.Were diagnostic tests or assessment methods and the results clearly described?* | **Y** |
| *5.Was the intervention(s) or treatment procedure(s) clearly described?* | **N** |
| *6.Was the post-intervention clinical condition clearly described?* | **N** |
| *7.Were adverse events (harms) or unanticipated events identified and described?* | **NA** |
| *8.Does the case report provide takeaway lessons?* | **Y** |

| **Study name: Vagner Fonseca 20201/5/15** | |
| --- | --- |
| **Items** | **Response options** |
| *1.Were patient’s demographic characteristics clearly described?* | **Y** |
| *2.Was the patient’s history clearly described and presented as a timeline?* | **Y** |
| *3.Was the current clinical condition of the patient on presentation clearly described?* | **Y** |
| *4.Were diagnostic tests or assessment methods and the results clearly described?* | **Y** |
| *5.Was the intervention(s) or treatment procedure(s) clearly described?* | **N** |
| *6.Was the post-intervention clinical condition clearly described?* | **N** |
| *7.Were adverse events (harms) or unanticipated events identified and described?* | **NA** |
| *8.Does the case report provide takeaway lessons?* | **Y** |

| **Study name: Yamilka Díaz 2021/7** | |
| --- | --- |
| **Items** | **Response options** |
| *1.Were patient’s demographic characteristics clearly described?* | **Y** |
| *2.Was the patient’s history clearly described and presented as a timeline?* | **Y** |
| *3.Was the current clinical condition of the patient on presentation clearly described?* | **Y** |
| *4.Were diagnostic tests or assessment methods and the results clearly described?* | **Y** |
| *5.Was the intervention(s) or treatment procedure(s) clearly described?* | **N** |
| *6.Was the post-intervention clinical condition clearly described?* | **N** |
| *7.Were adverse events (harms) or unanticipated events identified and described?* | **NA** |
| *8.Does the case report provide takeaway lessons?* | **Y** |

We arbitrarily defined the study at high quality if it clearly described clinical condition of the patients on presentation, diagnostic tests or assessment methods and the results, and all other items were assessed as Yes or NA; at low quality if it were not met both two criteria, regardless of assessment of other items; at moderate quality if did not meet criteria for high or low quality. We considered not applicable the seventh question of the case report checklist.

**eTable 3. JBI assessment results of cross-sectional studies**

| **Study name: Anna Jeffery-Smith 2020/11** | |
| --- | --- |
| **Items** | **Response options** |
| *1.* *Were the criteria for inclusion in the sample clearly defined?* | **Y** |
| *2.* *Were the study subjects and the setting described in detail?* | **Y** |
| *3.* *Was the exposure measured in a valid and reliable way?* | **Y** |
| *4.* *Were objective, standard criteria used for measurement of the condition?* | **Y** |
| *5.* *Were confounding factors identified?* | **N** |
| *6.* *Were strategies to deal with confounding factors stated?* | **N** |
| *7.* *Were the outcomes measured in a valid and reliable way?* | **Y** |
| *8.* *Was appropriate statistical analysis used?* | **Y** |

| **Study name: Philippe Brouqui 2021/03** | |
| --- | --- |
| **Items** | **Response options** |
| *1.* *Were the criteria for inclusion in the sample clearly defined?* | **Y** |
| *2.* *Were the study subjects and the setting described in detail?* | **Y** |
| *3.* *Was the exposure measured in a valid and reliable way?* | **Y** |
| *4.* *Were objective, standard criteria used for measurement of the condition?* | **Y** |
| *5.* *Were confounding factors identified?* | **N** |
| *6.* *Were strategies to deal with confounding factors stated?* | **N** |
| *7.* *Were the outcomes measured in a valid and reliable way?* | **N** |
| *8.* *Was appropriate statistical analysis used?* | **Y** |

We arbitrarily defined the study at high quality if it clearly described study subjects and the setting, identified and deal with the confounding factors, and all other items were assessed as Yes or NA; at low quality if it were not met all three criteria, regardless of assessment of other items; at moderate quality if it did not meet criteria for high or low quality.

**Table S4. JBI assessment results of case-control studies**

| **Study name: Letícia Adrielle dos Santos 2021/2/13** | |
| --- | --- |
| **Items** | **Response options** |
| 1. *Were the groups comparable other than the presence of disease in cases or the absence of disease in controls?* | **Y** |
| 1. *Were cases and controls matched appropriately?* | **Y** |
| 1. *Were the same criteria used for identification of cases and controls?* | **Y** |
| 1. *Was exposure measured in a standard, valid and reliable way?* | **Y** |
| 1. *Was exposure measured in the same way for cases and controls?* | **Y** |
| 1. *Were confounding factors identified?* | **N** |
| 1. *Were strategies to deal with confounding factors stated?* | **N** |
| 1. *Were outcomes assessed in a standard, valid and reliable way for cases?* | **Y** |
| 1. *Was the exposure period of interested long enough to be meaningful?* | **Y** |
| 1. *Was appropriate statistical analysis used?* | **Y** |

We arbitrarily defined the study at high quality if it appropriately matched cases and controls, exposure measured in the same way for cases and controls, identified and deal with the confounding factors, and all other items were assessed as Yes or NA; at low quality if it were not met all four criteria, regardless of assessment of other items; at moderate quality if it did not meet criteria for high or low quality.

**Table S5. NOS assessment results of cohort studies**

| **Study name:** Laith J. Abu-Raddad 2021/5 | |
| --- | --- |
| **Items** | **Response options** |
| Selection | |
| *Representativeness of the exposed cohort？* | *** |
| *Selection of the non exposed cohort* | *** |
| *Ascertainment of exposure* | *** |
| *Demonstration that outcome of interest was not present at start of study* | *** |
| Comparability | |
| *Comparability of cohorts on the basis of the design or analysis* | *** |
| Outcome | |
| *Assessment of outcome* | *** |
| *Was follow-up long enough for outcomes to occur* | *** |
| *Adequacy of follow up of cohorts* | *** |

Cohort studies with scores of 0-3, 4-6, 7-9 were, respectively, considered as low, moderate, and high quality.

**Table S6. Patients’ information**

| Study | Patient ID | First infection | | | | Reinfection | | | | Interval time between two symptoms of both infection( Days) | Outcome |
| --- | --- | --- | --- | --- | --- | --- | --- | --- | --- | --- | --- |
|  |  | Onset of symptoms | Nucleic acid test positive time | End time of symptoms | Nucleic acid test negative time | Onset of symptoms | Nucleic acid test positive time | End time of symptoms | Nucleic acid test negative time |  |  |
| Ana Lucia Frugis Yu | 1 | 2020/6/24 | 2020/6/29 | - | - | 2020/11/17 | 2020/11/23 | 2021/6/18 | - | - | - |
|  | 2 | 2020/8/12 | 2020/8/17 | - | - | 2021/1/1 | 2021/2/4 | 2021/6/18 | - | - | - |
| Antonio L. Aguilar-Shea | 3 | 2020/3/15 | 2020/3/- | 2020/4/15 | - | 2021/1/- | 2021/1/- | 2021/5/7 | - | - | Recovery |
| Belén Prado-Vivar | 4 | 2020/5/12 | 2020/5/20 | - | 2020/6/3 | 2020/7/20 | 2020/7/22 | 2020/9/8 | 2020/8/4 | - | - |
| Camila Malta Romano | 5 | 2020/9/25 | 2020/9/29 | - | - | 2021/2/- | 2020/2/4 | 2020/2/- | - |  | - |
| Carolina Kymie Vasques Nonaka | 6 | 2020/5/26 | 2020/6/1 | 2020/6/8 | - | 2020/10/26 | 2020/10/26 | 2021/4/23 | - | 140 | - |
| Daniela Loconsole | 7 | 2020/3/20 | 2020/3/21 | 2020/3/23 | - | 2021/1/8 | 2021/1/11 | - | 2021/1/23 | 291 | - |
| David Harrington | 8 | 2020/4/2 | - | - | - | 2020/12/11 | 2020/12/8 | - | - | - | Hospitalized |
| Federica Novazzi | 9 | 2020/12/31 | 2021/1/4 | - | 2021/1/23 | - | 2021/2/4 | - | - | - | Hospitalized |
|  | 10 |  | 2021/1/7 | - | - | - | 2021/2/2 | - | - | - | Hospitalized |
| Gabriela Sevillano | 11 | 2020/7/15 | 2020/7/20 | - | 2020/7/25 | 2020/10/26 | 2020/10/26 | - | 2021/2/2 | - | - |
| Jan Van Elslande | 12 | 2020/3/- | 2020/3/9 | 2020/4/4 | - | 2020/6/- | 2020/6/10 | - | - | - | Recovery |
| Jason D. Goldman | 13 | 2020/3/- | - | 2020/4/8 | - | 2020/7/- | - | - | - | - | - |
| Jayanthi Shastri | 14 | 2020/5/17 | 2020/5/15 | 20205/19 | 2020/5/19 | 2020/7/19 | 2020/7/19 | 2020/7/26 | 2020/7/29 | - | - |
|  | 15 | - | 2020/5/15 | - | 2020/5/18 | 2020/7/18 | 2020/7/18 | 2020/7/20 | 2020/7/25 | - | - |
|  | 16 | 2020/5/13 | 2020/5/14 | 2020/5/18 |  | 2020/7/7 | 2020/7/5 | 2020/7/28 |  | 50 | - |
|  | 17 | 2020/4/18 | 2020/4/20 | 2020/4/25 | 2020/4/23 | 2020/9/11 | 2020/9/14 | 2020/10/23 | 2020/9/18 | 139 | - |
| Jee-Soo Lee | 18 | 2020/3/5 | 2020/3/11 | 2020/3/25 | 2020/3/26 | 2020/4/5 | 2020/4/5 | 2020/4/9 | 2020/4/17 | 11 | - |
| Jonathan Klein | 19 | 2020/3/- | - | 2020/3/27* | - | - | - | - | - | - | Discharged |
| Juan David Ramírez | 20 | 2020/7/9 | - | - | 2020/8/3 | 2020/8/12 | 2020/8/12 | - | - | - | - |
| Juliana D. Siqueira | 21 | 2020/5/4 | 2020/5/4 | - | 2020/5/20 | 2020/8/16 | 2020/8/18 | - | - | - | Death |
| Kelvin Kai-Wang To | 22 | 2020/3/26 | 2020/3/26 | 2020/3/29 | 2020/4/14 | - | 2020/8/15 | - | - | - | - |
| LCDR Derek Larson | 23 | 2020/3/21 | 2020/3/20 | 2020/5/11 | - | 2020/5/24 | 2020/5/24 | - | - | 13 | - |
| Lucila Marquez | 24 | 2020/11/- | - | 2020/11/8* | - | 2020/1/- | - | - | - | - | Hospitalized |
| Mahesh S. Dhar | 25 | - | 2020/6/12 | - | 2020/6/27 | - | 2020/8/24 | - | - | - | - |
| Mariene R. Amorim | 26 | 2020/4/5 | 2020/4/9 | 2020/4/17 | 2020/4/22 | 2020/5/30 | 2020/6/10 | - | - | 43 | - |
|  | 27 | 2020/4/11 | 2020/4/14 | 2020/5/4 | 2020/9/2 | 2020/9/28 | 2020/10/1 | - | - | 147 | - |
|  | 28 | 2020/5/10 |  | 2020/5/21 | - | 2020/9/18 | - | 2020/9/29 | - | 120 | - |
|  | 29 | 2020/5/10 |  | 2020/5/20 | - | 2020/9/30 | - | 2020/10/9 | - | 133 | - |
| Mark I. Garvey | 30 | 2020/4/1 | 2020/4/1 | - | - | 2020/10/25 | 2020/11/2 | - | - | - | Death |
|  | 31 |  | 2020/5/23 | - | 2020/5/30 | 2020/12/- | 2021/1/2 | - | - | - | - |
|  | 32 | 2020/5/- | 2020/6/5 | - | - | - | 2021/1/28 | - | - | - | - |
| Mostafa Salehi-Vaziri（3） | 33 | 2020/7/1 | 2020/7/4 | - | - | 2020/11/9 | 2020/11/9 | - | - | - | - |
| Mostafa Salehi-Vazir | 34 | 2020/04/20 | - | - | - | 2020/07/17 |  | - | - | - | Recovered |
|  | 35 | 2020/04/04 | - | - | - | 2020/08/22 | - | - | - | - | Recovered |
|  | 36 | 2020/3/10 | - | - | - | 2020/7/4 |  | - | - | - | Recovered |
| Natalia Fintelman-Rodrigues | 37 | 2020/3/21 | 2020/3/23 | - | - | 2020/5/25 | 2020/5/29 | - | - | - | - |
|  | 38 | 2020/3/26 | 2020/3/24 | - | - | 2020/5/26 | 2020/5/29 | - | - | - | - |
|  | 39 | - | 2020/3/24 | - | - | 2020/5/27 | 2020/5/29 | - | - | - | - |
|  | 40 | 2020/3/31 | 2020/4/2 | - | - | 2020/5/30 | 2020/5/29 | - | - | - | - |
| Noémie Zucman | 41 | 2020/9/1 | - | - | 2020/12/- | 2020/1/- | 2020/1/- | - | - | - | - |
| Onkar Kulkarni | 42 | - | 2020/8/31 | - | - | 2020/11/14 | 2020/11/14 | - | - | - | - |
|  | 43 | 2020/11/4 | 2020/11/4 | 2020/11/5 | - | 2020/11/22 | 2020/11/22 | - | - | 17 | - |
| Pallavali R. Rani | 44 | - | 2020/7/25 | - | 2020/8/2 | - | 2020/9/10 | - | 2020/9/24 | - | - |
| Pauline Vetter | 45 | 2020/4/12 | 2020/4/10 | 2020/4/26 | - | 2020/10/30 | 2020/10/31 | - | - | 187 | - |
| Richard L Tillett | 46 | 2020/3/25 | 2020/4/18 | 2020/4/27 | - | 2020/5/28 | 2020/6/5 | - | - | 31 | Hospitalized |
| Thérèse Staub | 47 | - | - | - | - | 2020/2/- | 2020/2/16 | - | - | - | - |
|  | 48 | 2020/4/- | 2020/4/- | - | - | 2020/2/- | 2020/2/12 | - | - | - | - |
|  | 49 | - | - | - | - | 2020/2/12 | 2020/2/15 | - | - | - | - |
|  | 50 | 2020/11/- | 2020/11/18 | - | - | 2020/2/- | 2020/2/16 | - | - | - | - |
| Thomas Theo Brehm | 51 | 2020/3/18 | 2020/3/20 | 2020/3/25 | - | 2020/12/26 | 2020/12/27 | 2020/12/30 | 2021/1/11 | 276 | - |
| Vivek Gupta | 52 | - | 2020/5/5 | - | 2020/5/13 | - | 2020/8/21 | - | 14d | - | - |
|  | 53 | - | 2020/5/17 | - | 2020/5/27 | - | 2020/9/5 | - | 6d | - | - |
| Vagner Fonseca | 54 | 2020/5/18 | 2020/5/21 | - | 2020/12/21 | 2021/1/4 | 2021/1/6 | - | - | - | - |
| Yamilka D´ıaz | 55 | 2020/6/7 | - | - | - | 2020/12/5 |  | - | - | - | Recovery |
| Carlos Henrique Camargo | 56 | 2020/6/29 | 2020/6/29 | - | - | 2020/11/11 | 2020/11/11 | 2020/11/22 | 2020/11/16 | - | Discharged |
| Cinzia Borgogna | 57 | 2020/6/23 | 2020/6/23 | 2020/7/7 | 2020/7/28 | - | 2020/10/11 | 2020/7/14 | - | - | Death |
| Giuliana Scarpati | 58 | - | 2020/3/14 | - | - | 2021/1/26 | 2021/1/26 | 2021/7/13 | - | - | - |
| Marina Oliboni Moschetta | 59 | 2020/5/1 | 2020/5/1 | 2020/5/8 | - | 2021/1/1 | 2021/1/1 | 2021/3/20 | - | 238 | Recovery |
|  | 60 | 2020/8/1 | 2020/8/1 | 2020/8/3 | - | 2021/3/1 |  | 2021/3/20 | - | 210 | Recovery |
| Marta Massanella | 61 | 2020/3/23 | 2020/3/24 | 2020/4/15 | 2020/4/2 | 2020/8/28 | 2020/8/31 | 2020//9/18 | - | 135 | Discharged |

**Table S7. Viral mutations of reinfection cases**

| Study | Patients ID | Primary infection | | Reinfection | |
| --- | --- | --- | --- | --- | --- |
|  |  | Gene AND Amino acid | SNPs* | Gene AND Amino acid | SNPs |
| Ana Lucia Frugis Yu | 1 | **D614G** | - | **D614G,** V1176F | - |
|  | 2 | **D614G,** V1176F | - | **D614G,** V1176F, **E484K** | - |
| Abeer N. Alshukair | 3 | orflab:S2839, S:T302, ORF8:W45L, ORF8:L84S, N:S202N | C8782T, 19547(C)DEL, G22468T, G28027T, T28144C G28878A, G29742A | A405, orflab:T568I, orflab:S610L, orflab: F924, orflab:E942A, orflab: K2511, orflab: T2800P, orflab: T2967I, orflab: S4393A, orflab: P4715L orflab: D6374, **S:D614G,** E:T30I, M:T7I, ORF8:D35, ORF8:G66V, N:R203K, N:R203, N:G204R | C241T, C1480T, C1968T, С2094T, C3037T, A3090C, G7798A, A8663C, C9165T, T13442G, C14408T, C19386T, A23403G, C26333T, C26542T, C27998T, G28090T, G28881A, G28882A, G28883C |
| Belén Prado-Vivar | 4 | nsp12:P323L, nsp13:S485L, **S:D614G,** ORF3a:Q57H | C2113T, C3037T, C7765T, C14408T, C17690T, C18877T, A23403G, G25563T | nsp2:R218C, nsp13:I432T, nsp13:P504L, nsp13:Y541C, ORF8:L84S | C1457T, C8782T, T9445C, T17531C, C17747T, A17858G, C18060T, G18756T, A24694T, T28144C |
| Carolina Kymie Vasques Nonaka | 5 | ORFa:T1437I, S:G1219C, ORF6:I33T, N:I2927 | C4575T, G25217T, T27299C, T29148C | ORF1a:L3468V, ORF1a:L3930F, S:E484K, N:A119S, N:P207S, N:M234I | T25C, C100T, T3766C, T10667G, C11824T, C12053T, A12964G, G23012A, T28245C, C28253T, G28628T, C28892T, G28975T, C29754T |
| Cynthia Y. Tang | 6 | - | - | ORF1ab:D75E, NSP3:P971L, NSP12:P4715L, NSP14:F6158L, ORF8:V62L, ORF8:L84S, ORF7a:S81L, ORF10:I4L, **S:D614G,** N:R202K, N:G203R | - |
| Carlos Henrique Camargo | 7 | **S:D614G,** N:R203K, N:G204R, N:I292T, N:P383L, NS6:I33T | - | **S:D614G,** S:V1176F, N:R203K, N:G204R, NSP2:V577F, NSP7:L71F, NSP12:P323L, NSP16:R216N | - |
| Cinzia Borgogna | 8 | ORF1b:P314L, S:A846V, ORF3a:R30S, ORF3a:R30L, N:R203K, N:G204R, ORF10 | - | ORF1b:P314L, **S:D614G** | - |
| Daniela Loconsole | 9 | **S:D614G,** N:G204R, N:R203K, NSP12: P323L | - | **S:D614G,** S:A222V, N:A222V, NS8:P30L, NSP3: N1116S, NSP3:T611I, NSP6:A54S, NSP12:P323L, NSP14:A323G | - |
| David Harrington | 10 | - | - | S:N501Y, S:A570D, **S:D614G,** S:P681H, S:T761I, S:S982A, S:D1118H. | - |
| Federica Novazzi | 11 | - | N501Y, A570D | - | N501Y, A570D |
| Giuliana Scarpati | 12 | 5'UTR:241, NSP1:V60V, NSP2:R27C, NSP3:F106F, NSP12b:P314L, NSP12b:D815G, NSP14:A267A, NSP14:A323V, NSP15:L245L, M:L93L, ORF8:H17H, N:A220V, ORF10:V30L | - | 5'UTR:241, NSP1:V60V, NSP2:E37E, NSP3:F106F, NSP3:T1189T, NSP4:A307V, NSP12b:P314L, NSP14:A267A, NSP16:A199A, S:A222V, M:L93L, ORF8:H17H, N:A220V, ORF10:V30L | - |
| Jason D. Goldman | 13 | - | MT252824, C8782T, T28144C | - | C3037T, C14408T, A23403G, G25563T |
| Jayanthi Shastri | 14 | - | - | - | - |
|  | 15 | **S:D614G,** S:Q677H | N1123N | - | A1812D |
|  | 16 | - | - | - | - |
|  | 17 | F924F | - | - | - |
| Jee-Soo Lee | 18 | ORF1ab:Q87D, ORF1ab:M951I, ORF1ab:N1181=, ORF1ab:T1334A, ORF1ab:L37F, ORF1ab:Y455=, ORF3a:T223I, ORF3a:G251V | - | **S:D614G,** nsp:F106F, P323L, ORF3a:Q57H, nsp1:R124C, nsp2:T85I, nsp3:L744F, nsp3:L1035F, nsp4:V407fs, nsp4:S481L, nsp9:L42P, nsp13:T115I, N:T165= | - |
| Jonathan Klein | 19 | **S:D614G** | - | **S:D614G,** S:A1078S | - |
| Juan David Ramírez | 20 | - | - | ORF1ab:C1059T, ORF1ab:G3483A, S:T23443C, N:G28881A, N:G28882A, N:G28883C | - |
| Marta Massanella | 21 | - | - | S:L18F, S:A222V, **S:D614G,** nsp12:P323L, N:A220V, ORF14:L67F, ORF10:V30L | - |
| Mahesh S. Dhar | 22 | - | C241T, A9419G, A23403G | - | C241T, A9419G, A23403G |
| Mariene R. Amorim | 23 | **D614G,** V1176F | - | **D614G,** V1176F | - |
|  | 24 | - | - | **D614G** | - |
|  | 25 | - | - | - | - |
|  | 26 | - | - | V1176F | - |
| Mostafa Salehi-Vaziri(3) | 27 | ORF1ab, NSP1:F 106 F ORF1ab, NSP3:T 2685 P ORF1ab, NSP12: P 323 L,  ORF1ab, NSP14:L 280 L ORF1ab, NSP14:I 210 del S:D294D, **S:D614G,** NS3:Q57H, M:Y71Y, N:S194L | - | ORF1ab, NSP1:E 37 D ORF1ab, NSP1:F 106 F ORF1ab, NSP3:T 2007 I ORF1ab, NSP3:T 2685 P ORF1ab, NSP12: R 52 K ORF1ab, NSP12:P 323 L ORF1ab, NSP14:L 280 L ORF1ab, NSP14:I 210 del S:D294D, **S:D614G,** NS3:Q57H, M:F110F, M:Y71Y, N:S194L, N:Y268Y | - |
| Mostafa Salehi-Vaziri | 28 | - | - | **D614G** | - |
|  | 29 | L139L | - | L139L | - |
|  | 30 | - | - | **D614G** | - |
| Natalia Fintelman-Rodrigues | 31 | **D614G** | - | **D614G** | - |
| Noémie Zucman | 32 | - | - | S:D80A, **S:E484K,** S:N501Y | - |
| Onkar Kulkarni | 33 | - | - | - | C3211A, C4002T, C6040T, A6498G, A8567G, C8917T, C20032T, G25855T, G27952T |
|  | 34 | - | - | - | G61T, C6040T, C14408T, C22006T, A23403G, G28881A |
| Pallavali R. Rani | 35 | C241T, C3037T, C3267T(T1001I), C11408T(P314L), C18877T, C21034T(L2523F), C22444T, T22882G(**N440K**), A23403G(**D614G**), 25563T(Q57H), G26173T(E261),  C26735T, G28183T(S97I), T28277C(S2P), C28854T(S194L) |  | C222T, C241T, C3037T, C3267T(T1001I), G11410A, C11408T(P314L), C18877T, C21034T(L2523F), C22444T, T22882G(**N440K**), 23403G**(D614G),** 25563T(Q57H), C26735T, G28183T(S97I), T28277C(S2P), C28320T (T16M), C28854T(S194L) |  |
| Thomas Theo Brehm | 36 | ORF1a: G392D, ORF1a: A876T | G1440A, G2891A | ORF1b: T17I, ORF1b: P314L, S: A222V, **S: D614G,** ORF3a:S165F, ORF9(N):A220V, ORF10: V30L | G204T, C241T, T445C, 3037T, T3592C, C6286T, C12076T, C13517T, C14408T, C19386T, G21255C, C22227T, A23403G,  C25886T, C26801G, C27944T, C28932T, G29645T |
| Vivek Gupta | 37 | - | C241T, T1947C, C6445T, G11383A, T11408C, G17584T, C18877T, A23403G, C23934T, C25207T, G25563T, C26456T, C26735T | - | C241T, C2367T, C13730T, C14408T, G17584T, C18877T, G19109T, T22882G, A23403G, C23929T, G24794A, G25563T, C26735T, C29215T |
| Vagner Fonseca | 38 | ORF1ab:P4715L, ORF1ab:M6078I, **S:D614G,** S:V1176F, Nucleocapsid:R203K, Nucleocapsid:G204R, ORF14:G50N |  | ORF1ab:T265I, ORF1ab:M2606I, ORF1ab:L3352F, ORF1ab:P4075S, ORF1ab:A4489V, ORF1ab:P4715L, ORF1ab:N6054D, ORF1ab:T6938I, ORF1ab:R7014C, ORF1ab:T265I, **S:D614G,** ORF3a:Q57H, ORF3a:G172V, ORF8: S24L, Nucleocapsid: P67S |  |
| Yamilka D´ıaz | 39 | nsp4:F308Y, ORF3a:G196V, ORF8:L84S | T9477A, C12815T, C14805T, G25979T, T28144C, C28657T | nsp1:L4F, nsp3:T725I, nsp3:K839E, nsp4:F308Y, nsp4:T492I, nsp6:H11Q, Helicase:P77L, Helicase:E319D, **S:D614G,** S:**L452R**, ORF3a:S74F, ORF3a:G196V, ORF8:L84S, N:S197L, N:M234I, N:P365S, N:P383L | C275T,C1567T,G2272A,C4543T,C4893T,A5234G,C8782T,T9477A,C10029T,C11005A,C12815T,C16466T,T16857C,G17193T,T18417C,T22917G,A23403G,G24697A,C25613T,G25979T,C26681T,T28144C,C28657T, |

“SNPs*”: Single-nucleotide polymorphisms
